# Systematic Screening of Penetratin’s Protein Targets by Yeast Proteome Microarrays

**DOI:** 10.3390/ijms23020712

**Published:** 2022-01-10

**Authors:** Pramod Shah, Chien-Sheng Chen

**Affiliations:** 1 Institute of Systems Biology and Bioinformatics, Department of Biomedical Sciences and Engineering, College of Health Sciences and Technology, National Central University, Jhongli 300, Taiwan; prokonp@gmail.com; 2 Department of Nutritional Science, Fu Jen Catholic University, New Taipei City 242, Taiwan; 3 Department of Food Safety/Hygiene and Risk Management, College of Medicine, National Cheng Kung University, Tainan 701, Taiwan

**Keywords:** penetratin, cell penetrating peptide (CPP), antimicrobial peptide (AMP), antifungal activity, proteome microarray, target identification, high-throughput screening

## Abstract

Cell-penetrating peptides (CPPs) have distinct properties to translocate across cell envelope. The key property of CPPs to translocation with attached molecules has been utilized as vehicles for the delivery of several potential drug candidates that illustrate the significant effect in in-vitro experiment but fail in in-vivo experiment due to selectively permeable nature of cell envelop. Penetratin, a well-known CPP identified from the third α-helix of Antennapedia homeodomain of Drosophila, has been widely used and studied for the delivery of bioactive molecules to treat cancers, stroke, and infections caused by pathogenic organisms. Few studies have demonstrated that penetratin directly possesses antimicrobial activities against bacterial and fungal pathogens; however, the mechanism is unknown. In this study, we have utilized the power of high-throughput *Saccharomyces cerevisiae* proteome microarrays to screen all the potential protein targets of penetratin. *Saccharomyces cerevisiae* proteome microarrays assays of penetratin followed by statistical analysis depicted 123 *Saccharomyces cerevisiae* proteins as the protein targets of penetratin out of ~5800 *Saccharomyces cerevisiae* proteins. To understand the target patterns of penetratin, enrichment analyses were conducted using 123 protein targets. In biological process: ribonucleoprotein complex biogenesis, nucleic acid metabolic process, actin filament-based process, transcription, DNA-templated, and negative regulation of gene expression are a few significantly enriched terms. Cytoplasm, nucleus, and cell-organelles are enriched terms for cellular component. Protein-protein interactions network depicted ribonucleoprotein complex biogenesis, cortical cytoskeleton, and histone binding, which represent the major enriched terms for the 123 protein targets of penetratin. We also compared the protein targets of penetratin and intracellular protein targets of antifungal AMPs (Lfcin B, Histatin-5, and Sub-5). The comparison results showed few unique proteins between penetratin and AMPs. Nucleic acid metabolic process and cellular component disassembly were the common enrichment terms for penetratin and three AMPs. Penetratin shows unique enrichment items that are related to DNA biological process. Moreover, motif enrichment analysis depicted different enriched motifs in the protein targets of penetratin, LfcinB, Histatin-5, and Sub-5.

## 1. Introduction

Anti-microbial peptides (AMPs), produced throughout the living organisms, provide the first line of rapid defense against invading pathogens [1]. Generally, AMPs are positively charged short peptides (usually >50 residues) with a wide range of antimicrobial activities against pathogenic bacteria, fungi, viruses, and even cancer cells [2,3]. Non-specific mechanism of action, low required dose, activities against multidrug-resistant pathogens, and hard-to-develop resistance for AMPs, together with the decline in discovery of antibiotics have attracted significant attention toward APMs for the development of alternative therapeutics; few AMPs are already in clinical trials [4,5,6,7].

Owing to its positive charge, AMPs target negatively charged phospholipid membranes. Based on membrane activity, AMPs are categorized into two major classes: one that targets membrane (cause membrane disruption and lead to cell death) and the other that penetrate across membranes without disrupting membrane (target intracellular molecules) [8]. The intracellular targeting AMPs with the ability to cross membrane are studied as transport vectors to deliver the bioactive substances inside the cells [9,10]. Cell-penetrating peptides (CPPs) are small cationic peptides with membrane translocation ability widely used for the internalization of attached cargo molecules [11,12,13]. CPPs translocate themselves as well as with attached cargo molecules, hence facilitating the transportation of bioactive cargo molecules like plasmid, small interfering RNAs, proteins and peptides, drugs, and nanoparticles [14,15] inside the cell for the diagnosis and treatment of diseases, like cancer and strokes [16,17,18,19,20,21]. CPPs can translocation across different types of cells with low cytotoxicity [22]. The mechanism of CPPs internalization mostly occurs through endocytosis followed by endosomal escape [15].

CPPs are also employed to transport antimicrobial drugs that target intracellular pathogens [23,24]. Along with internalization and cargo delivery, CPPs themselves are reported to have antimicrobial activities against bacteria and fungi [25].

Penetratin, a well-known CPP with 16 amino acids (43–58 residues) and a net positive charge of +7, was identified from the third α-helix of Antennapedia homeodomain (DNA-binding motif) of Drosophila homeoprotein [26,27,28]. Penetratin was demonstrated to internalize across the membrane in an energy-independent manner at both 4 °C and 37 °C. Further, the intact penetratin was recovered from the cytoplasm and nucleus [27,28].

Further studies demonstrated the translocation of penetratin through the receptor-independent manner [28], charge-dependent [29], energy-dependent, and lipid raft-mediated endocytic transportation [30]. In Chinese Hamster Ovary cells, translocation of penetratin was observed through 30% direct translocation and 70% endocytosis [31]. While earlier Biophysical studies demonstrated penetratin binding to anionic phospholipids [29] later it was concluded that a mechanism like endocytosis is responsible for the internalization of penetratin across the membrane [32]. Moreover, penetratin/lipid interaction is mostly concentration-dependent, where penetratin exerts α-helical conformation at low concentration and β-sheet at high concentration [26]. The change in conformation from α-helices to β-sheet increases the translocation ability of CPPs [33]. In fungal cell, penetratin exhibited >80% of translocation with direct and endocytosis translocation [34].

Penetratin is widely studied for its cargo delivery [35] and is also reported to exert antibacterial [36] and antifungal activities against *Candida albicans* and *Cryptococcus neoformans* [37,38]. 50% minimal inhibitory concentration (i.e., MIC 50) of penetratin against *Candida albicans* and *Candida glabrata* were reported as 1 µM and 2 µM, respectively [34]. Also, penetratin demonstrated minimal hemolytic effect on bovine erythrocytes at a concentration of 50 µM [39]. Biological and biophysical studies on penetratin have provided meaningful insight into translocation, cargo delivery and antimicrobial activities, however the antimicrobial mechanism of action and the target are unclear. In this study, we have deployed *Saccharomyces cerevisiae* proteome microarrays to systematically screen the entire fungal protein targets of penetratin. *Saccharomyces cerevisiae* proteome microarrays have provided a systematical screening platform to identify the protein targets of antifungal AMPs [40,41]. The identified protein targets of penetratin were subjected to bioinformatics tools for enrichment analysis. Moreover, a comparative study was performed to understand the mechanism of penetratin and antifungal AMPs.

## 2. Results

### 2.1. Yeast Proteome Microarrays Assays

**Figure 1:**
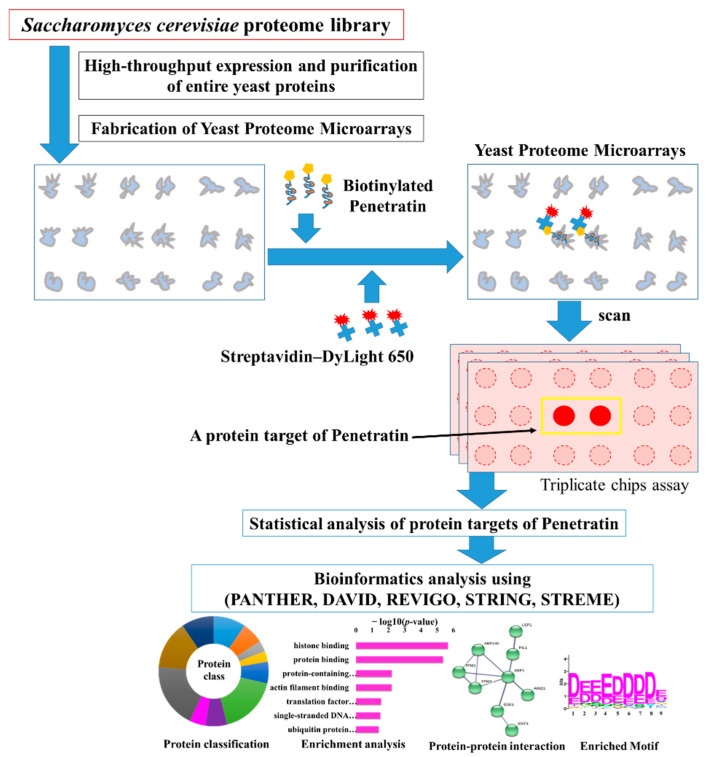
Schematic diagram of this study. For the screening of the protein targets of penetratin, biotinylated penetratin was probed on yeast proteome microarrays followed by streptavidin-DyLight 650 and anti-His antibody-DyLight 550. For simplicity, His tag and anti-His antibody signal of the yeast protein are not shown. The yeast proteome microarrays were scanned and the interacting signals of penentratin with yeast proteins partners were obtained. Data from triplicate yeast proteome microarrays assays of penetratin were statistically analyzed following cutoff parameters to identify the protein targets of penetratin. These identified protein targets were subjected to several bioinformatics tools, like PANTHER, DAVID, REVIGO, STRING, and STREME databases to understand the binding pattern through protein classification, enrichment in gene ontology, protein–protein interaction network, and enriched motif.

Yeast proteome microarrays containing ~5800 individually purified *Saccharomyces cerevisiae* proteins spotted (in duplicate) on an aldehyde-coated glass slide were used to systematically screen the protein targets of penetratin. Yeast proteome microarrays is an unbiased high-throughput screening platform that provides the interacting partners in a single assay. For yeast proteome microarray assay of penetratin, biotinylated penetratin was probed on yeast proteome microarray followed by the probing of streptavidin conjugated DyLight 650 and anti-His antibody conjugated DyLight 550 probing which was later scanned to obtain the binding signals. Streptavidin-DyLight 650 detected the signal of biotinylated penetratin bound to yeast proteins on the yeast chips. On the other hand, anti-His antibody-DyLight 550 detected the signal of His-tag on yeast protein, which also represented the total quantity of individual yeast protein on yeast chip. Triplicate yeast chips assays of penetratin were conducted and data were statistically analyzed to identify the yeast protein targets of penetratin. The identified yeast protein targets of penetratin were analyzed with bioinformatics tools (PANTHER, DAVID, REVIGO, STRING, STREME) to identify enrichment in protein classification, Gene Ontology (GO), protein domain family (PFAM), protein-protein interaction, and motif. Figure 1 depicts the schematic diagram of this study. As duplicate spots of individual proteins are spotted on yeast proteome microarrays, a protein target of penetratin on yeast chips is represented by duplicate red spots inside a yellow rectangular box (Figure 1). 

### 2.2. Statistical Analysis of Protein Targets of Penetratin

The scanned results of triplicate yeast proteome microarrays assay of penetratin were opened using GenePix Pro software. GenePix Pro software allowed us to identify and fix the intensity of each protein spot on yeast chips. After alignment of each yeast protein spot with yeast protein name, the data were exported as GenePix Pro Results (GPR) files. Three GPR files of the triplicate yeast chip assays were opened in excel files. Triplicate data were analyzed together using statistical cutoff parameters to identify the protein targets of penetratin. The statistical paraments used are median scaling normalization for sample (i.e., penetratin binding signals) and control (i.e., anti-His antibody signals). Signal intensity cutoff of >100 was applied for the sample, coefficient of variable (CV) between the duplicate spots for the sample were selected <0.05, the signal ratio between sample and control was >0.5, and then a local cutoff parameter of sample signal > mean plus two standard deviations (SD) was used to identify the yeast protein targets of penetratin. Finally, the duplicate spots of each identified protein from the triplicate yeast chips were confirmed to have outstanding signal than background. Using these all criteria, 123 yeast proteins were identified as the protein targets of penetratin. Protein ID, uniprot ID, protein name, and subcellular location of each identified protein target of penetratin are displayed in Supplementary Table S1.

**Figure 2:**
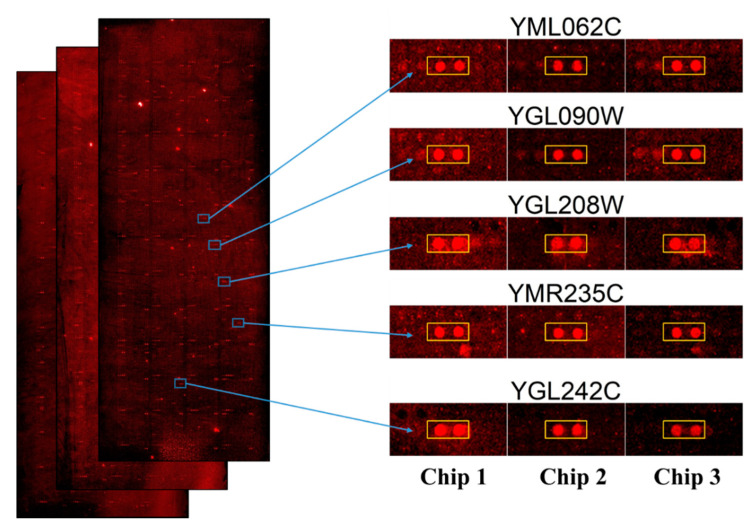
Image of protein targets of penetratin identified from yeast proteome microarrays. Entire yeast proteome microarrays probed with penetratin followed by streptavidin-DyLight 650 (**left**); the enlarged images of each representative protein targets (in duplicate red spot enclosed inside the yellow box) of penetratin from the triplicate yeast proteome microarrays (**right**).

Figure 2 depicts the scanned images of yeast proteome microarrays probed with penetratin. The entire yeast chips probed with penetratin followed by streptavidin-DyLight 650 (in triplicate) are shown in Figure 2 (left). The enlarge image of the representative protein targets of penetratin (in duplicate red spots enclosed inside the yellow box) identified from the three independent yeast chips is shown in Figure 2 (right). Supplementary Figure S1 displays the enlarged image of all the 123 yeast protein targets of penetratin from triplicate yeast chips.

### 2.3. Protein Classification of the Protein Targets of Penetratin

**Figure 3:**
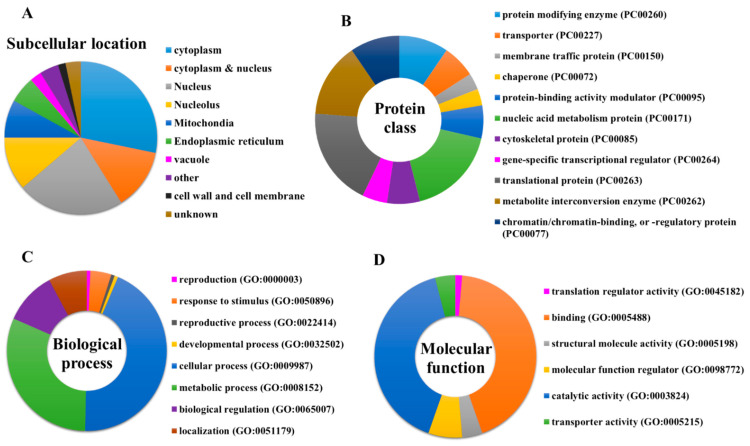
(**A**) Subcellular location of entire protein targets of penetratin. PANTHER classification system to classify the identified protein targets of penetratin into (**B**) protein class, (**C**) biological process, and (**D**) molecular function.

UniProt database was used to obtain the protein name, function, subcellular location, and FASTA sequence of all the 123 identified protein targets of penetratin [42]. Figure 3A shows the subcellular location of 123 identified protein targets of penetratin. The result indicates that the identified protein targets of penetratin mostly reside inside the cytoplasm and nucleus. This is consistent with the previous result where intact penetratin was recovered from the cytoplasm and nucleus [27]. Along with the majority of targeting cytoplasmic and nucleus proteins, penetratin also targets proteins residing in the nucleolus, mitochondria, and endoplasmic reticulum.

To determine the classification of the newly identified protein targets of penetratin, these proteins were subjected to PANTHER classification system [43]. The results of protein classification are shown as protein class (Figure 3B), biological process (Figure 3C), and molecular function (Figure 3D), respectively. The protein targets of penetratin were classified into 11 protein classes of which “nucleic acid metabolism protein (PC00171)”, “translational protein (PC00263)”, and “metabolite interconversion enzyme (PC00262)” are the top three classes (Figure 3B). In terms of biological process, 8 groups of biological processes were identified where “cellular process (GO:0009987)” and “metabolic process (GO:0008152)” were the two top groups (Figure 3C). Six groups were noted in the molecular function categories for the protein targets of penetratin. The two top groups in molecular function are “binding (GO:0005488)” and “catalytic activity (GO:0003824)”.

### 2.4. Enrichment Analysis in Gene Ontology for the Protein Targets of Penetratin

To identify the enrichment categories in Gene Ontology (GO) classes of biological process, molecular function, and cellular component, DAVID database version 6.8 was used [44,45]. The stringent *p*-value cutoff of <0.05 was applied to select the significantly enriched terms in all the GO categories.

Under GO biological process category, DAVID provided a long list of 108 enriched terms with *p*-value cutoff of <0.05. The top 20 enrichment terms of GO biological process for the protein targets of penetratin are listed in Table 1. The entire enrichment items in GO biological process for the protein targets of penetratin are depicted in Supplementary Table S2. Along with enriched terms in biological process, Table 1 also displays GO ID for the term, *p*-value, the number of protein targets of penetratin in the enriched category, and the total number of yeast proteins that belong to the enriched category. The enriched terms “cellular component biogenesis”, “cellular component organization or biogenesis” and “cellular component assembly” depicted the biological role of the identified protein targets of penetratin in the cellular component organization. Ribosome assembly and biogenesis were also highly enriched in GO biological process with the enriched terms: “ribosome biogenesis”, “ribosomal large subunit biogenesis”, “ribosomal large subunit assembly”, and “ribosome assembly”. Several terms for ribonucleoprotein complex biogenesis, like “ribonucleoprotein complex biogenesis”, “ribonucleoprotein complex subunit organization” and “ribonucleoprotein complex assembly” also depicted the role of the protein target of penetratin in ribonucleoprotein complex organization. Moreover, several GO terms were enriched for actin filament organization.

The GO enrichment result in biological process (BP) obtained from DAVID database by selecting “GOTERM_BP_ALL (i.e., all level)” includes all the possible terms in GO hierarchy (i.e., sibling terms) and inheritance related (child and parent terms), thus providing a long list of enriched items. These redundant lists of GO enrichments cause difficulty in the result interpretation. Hence, an online REVIGO platform was used to obtain a single representative GO term for the redundant enrichment terms (i.e., REVIGO remove the semantically similar GO terms) [46]. REVIGO analysis with a cutoff of 0.5 identified 29 non-redundant enriched items from the 108 enriched terms GO biological process (displayed in Table 2 with the degree of frequency, uniqueness, and dispensability). The GO items: “cellular component biogenesis”, “cellular component organization or biogenesis”, “cellular component organization”, “regulation of cellular component biogenesis” and “cellular component disassembly” are non-redundant terms; thus, they were not removed from the biological process that indicate assembly, arrangement, and disassembly of a cellular component. Several terms related to the ribosome in Table 1 i.e., “ribosome biogenesis”, “ribosomal large subunit biogenesis”, “ribosomal large subunit assembly”, and “ribosome assembly” were removed and only “ribosomal large subunit biogenesis” was retained, which represents all the other ribosomal process. Moreover, GO terms related to ribonucleoprotein organization i.e., “ribonucleoprotein complex biogenesis”, “ribonucleoprotein complex subunit organization” and “ribonucleoprotein complex assembly” in Table 1 were partly retained in Table 2 with terms “ribonucleoprotein complex biogenesis” and “ribonucleoprotein complex subunit organization”. The biological process terms that include “ribosome large subunit biogenesis” is a function of “ribonucleoprotein complex biogenesis”, which is a function of “cellular component biogenesis” which is a function of “cellular component organization” which is further a function of “cellular process”. Hence, “cellular process” was depicted as a top group in PANTHER analysis of the biological process.

Protein targets of penetratin show significant enrichment in biological processes related to organization or biogenesis of cellular component, ribonucleoprotein complex, ribosomal large subunit, protein-containing complex subunit (also known as “macromolecular complex subunit”), organelle, chromosome, and actin cytoskeleton. The “metabolic process” depicted as the second top group of biological process in Figure 3 is associated with the different metabolic processes that involved “nucleic acid metabolic process”, “heterocycle metabolic process”, “cellular aromatic compound metabolic process”, “organic cyclic compound metabolic process” and “cellular macromolecule metabolic process” (Table 2).

**Figure 4:**
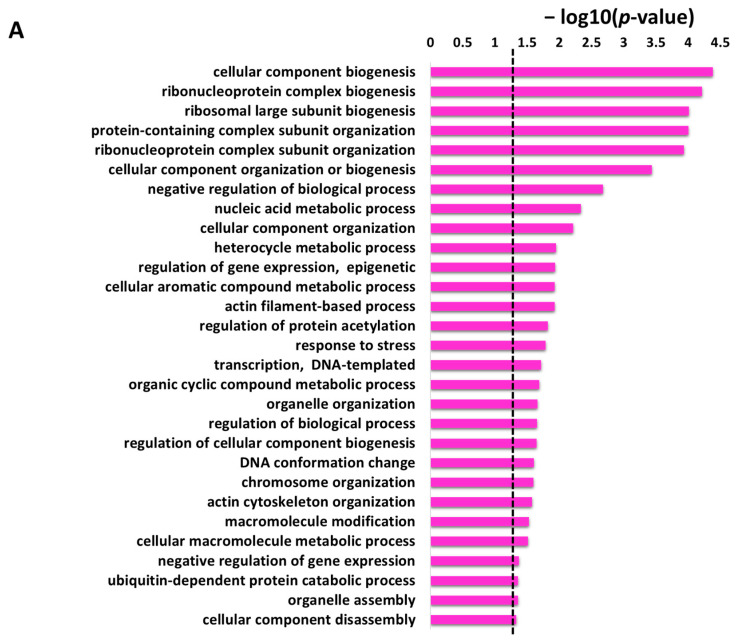

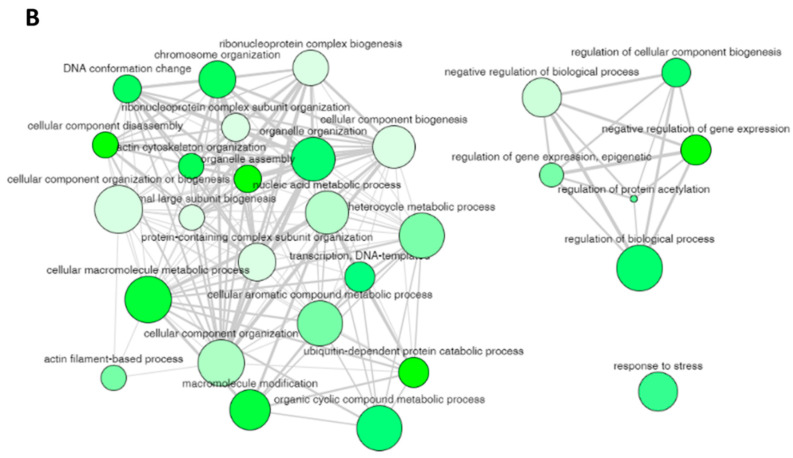
REVIGO analysis for the non-redundant enrichment items in the biological process. (**A**) The 29 non-redundant enrichment items in the biological process that were retained in REVIGO analysis obtained from DAVID database with the corresponding *p*-value of these items (the dotted line represents *p*-value = 0.05). (**B**) Cluster between the non-redundant items with similar biological process. The non-redundant terms are represented by bubbles and the color of the bubbles illustrate the user-provided *p*-value (lowest *p*-value is light green color whereas the highest *p*-value = 0.05 has dark green color). The size of the bubbles indicates the frequency of each GO term identified in the Gene Ontology Annotation database (larger bubbles are for more general terms). The width of the line connecting the bubbles represents the degree of similarity.

The non-redundant enriched items identified by REVIGO for the enriched items in GO biological process are illustrated in Figure 4A with a *p*-value cutoff (the dotted line represents the *p*-value = 0.05). These enrichment terms in the biological process depicted several terms related to cellular and metabolic processes. The identified 29 non-redundant items in the biological process (Table 2) can still have some degree of similarity (as we used only a cutoff parameter of 0.5) and these terms can be cluster together to represent specific process. The 29 non-redundant biological process terms were clusters by REVIGO database and the results are displayed in Figure 4B. The non-redundant enrichment items are illustrated by bubbles, and their size implies the frequency of the items identified in the Gene Ontology Annotation database (shown in the “Frequency” column in Table 2). High frequency represented by larger bubbles is the more general term, whereas low frequency (smaller size of bubbles) indicates a more specific term. For example, the term “regulation of protein acetylation” with the lowest frequency of 0.1823 (Table 2) represents a specific term and is shown by the smallest bubble in Figure 4B. The color of the bubbles (light to dark green) indicates the corresponding *p*-values of the enriched items, i.e., term with a *p*-value close to 0.05, is shown in dark green. The bubble representing the non-redundant enrichment items is connected by a line based on the similarity of the two terms (Figure 4B). The width of the line connecting the bubbles indicates the degree of similarity. The items targeting similar biological processes are clustered together. Two clusters are visualized for the 29 enriched terms in the biological process for the protein targets of penetratin (Figure 4B). One cluster contains GO terms related to component biogenesis or organization and metabolic process whereas the other cluster consists of GO terms related to gene expression and biological process. The non-redundant term “response to stress” has no similarity to these two clusters.

The significant enriched items identified in the GO category of cellular component for the protein targets of penetratin are depicted in Table 3. The stringent *p*-value cutoff of <0.05 was applied for the selection of significantly enriched terms in the cellular component. The protein targets of penetratin that contribute to the significant enrichment term in the cellular component are depicted in column “Hit in this category”, and the proteins from entire *Saccharomyces cerevisiae* that belong to this specific item are shown in column “Total gene in this category”. The results indicate the protein targets of penetratin localize inside the cytoplasm and is sub-localize in organelles like “non-membrane-bounded organelle”, “cortical cytoskeleton”, “nucleus”, “nucleolus”, “membrane-enclosed lumen”, “actin cytoskeleton” and others.

The enriched GO terms in the cellular component with their corresponding *p*-value were analyzed using REVIGO to identify the non-redundant enrichment items. REVIGO identified 11 non-redundant enrichment items (Table 4) from the list of 31 enrichment items (Table 3). The protein targets of penetratin show cellular component enrichment in “cytoplasmic region”, “nucleus”, and “nucleolus” and sub-localization in “non-membrane-bounded organelle”, “membrane-enclosed lumen”, and others like “cortical cytoskeleton”, etc. (Table 4). Table 4 also displays frequency, uniqueness, and dispensability of each non-redundant enrichment item in the cellular component for the protein targets of penetratin.

**Figure 5:**
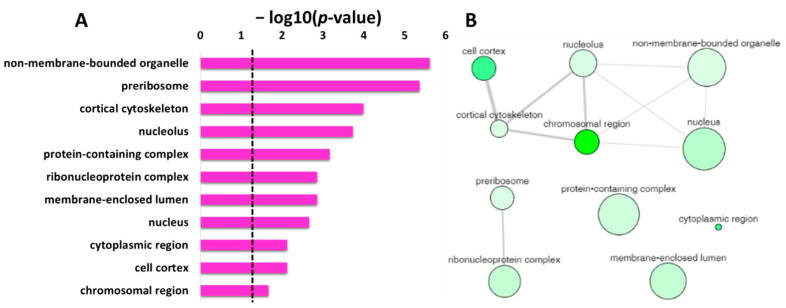
REVIGO analysis for the non-redundant enrichment items in the GO cellular component. (**A**) list of non-redundant enrichment items in cellular component with corresponding *p*-value (the dotted line represents *p*-value = 0.05). (**B**) The graph-based visualization of clusters for similar non-redundant items in cellular component. The non-redundant terms are represented by bubble; the color of the bubbles from light to dark green illustrates the user-provided *p*-value from lowest to highest *p*-value = 0.05. The size of the bubbles indicates the frequency of each GO term (larger bubbles are for more general terms) and the width of the line connecting the bubbles represents the degree of similarity.

Figure 5A displays the non-redundant enriched terms in GO category of cellular component for the protein targets of penetratin obtained by REVIGO. These non-redundant items were clustered based on the similarity, as depicted in interactive graphic visualization in Figure 5B. The size of the bubbles corresponds to the frequency of each item in Table 4, and the color of the bubbles from the light to dark green indicates the low to high *p*-value. The width of the line represents the degree of similarity between the two enrichment items. Based on the similarity in co-localization of the protein inside the yeast cell, a cluster is observed for “cell cortex”, “cortical cytoskeleton”, “non-membrane-bounded organelle”, “nucleus”, “chromosomal region” and “cortical cytoskeleton”. Another cluster is observed for “preribosome” and “ribonucleoprotein complex”, which is obvious as these terms are related to protein binding to RNA. The three items “protein-containing complex”, “membrane-enclosed lumen”, and “cytoplasmic region” were independent in Figure 5B, indicating these terms are not related to the two identified clusters.

DAVID database was used to obtain the list of GO enrichment terms in molecular function category for the protein targets of penetratin (Table 5). To obtain only significant terms, *p*-value cutoff of lower than (<) 0.05 was applied. Table 5 shows the protein targets of penetratin are significantly enriched in “histone binding” and “protein binding” terms along with enrichment in “actin filament binding” and others. This demonstrates the identified protein targets of penetratin affect the proteins that function in protein and protein complex, nucleic acid (DNA and RNA) and histone, actin filament, and ubiquitin-related activities.

The list of enrichment GO terms in molecular function for the protein targets of penetratin (Table 5) were analyzed by REVIGO database. The results as depicted in Table 6 show non-redundant terms in the identified enriched terms for molecular function. For the enriched terms “ubiquitin protein ligase activity” and “ubiquitin-like protein ligase activity”, the term “ubiquitin protein ligase activity” is a more specific term than “ubiquitin-like protein ligase activity” in the hierarchical ancestor chart for GO:0061630 in EMBL-EBI database (https://www.ebi.ac.uk/ (accessed on 5 November 2021)). Hence, “ubiquitin protein ligase activity” was retained, representing a non-redundant term in both the enriched terms. The enrichment results in Table 6 are similar to Figure 3D that illustrate the protein targets of penetratin are significantly enriched in “actin filament binding” (i.e., structural molecular activity in Figure 3D), “ubiquitin protein ligase activity” (i.e., catalytic activity in Figure 3D) and so on. The enriched item “macromolecular complex binding” (in Table 5), is replaced with “protein-containing complex binding” (in Table 6).

**Figure 6:**
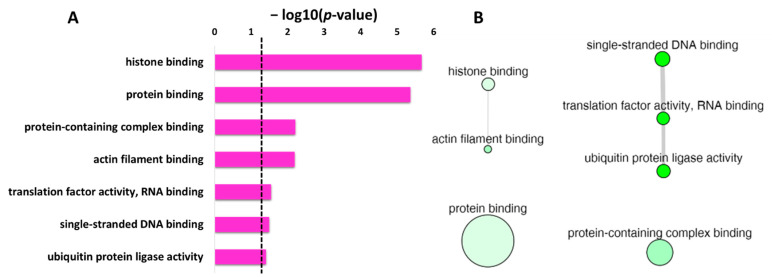
REVIGO analysis for the non-redundant enrichment items in molecular function for the protein targets of penetratin. (**A**) list of non-redundant enrichment items in molecular function category with corresponding *p*-value (the dotted line represents *p*-value = 0.05). (**B**) The graph-based visualization shows clusters between similar non-redundant items in molecular function. Non-redundant terms are represented by bubbles, the color of bubble from light to dark green illustrates the *p*-value from lowest to highest, the size of the bubbles indicates the frequency of each GO term, and the width of the line connecting the bubbles represent the degree of similarity.

The non-redundant enrichment items in the GO category of molecular function with *p*-value are depicted in Figure 6A. Based on the degree of similarities between the enriched terms, these terms were clustered as depicted in Figure 6B. The “protein binding” with the second lowest *p*-value but the highest frequency value of 40.91 (Table 6), is shown by the largest bubble. Whereas “histone binding”, the lowest *p*-value with a frequency of 0.89 (Table 6), is represented by a small size bubble. The enrichment terms “single-stranded DNA binding”, “translation factor activity, RNA binding” and “ubiquitin protein ligase activity” though non-redundant enrichment terms, have similarity in the function, and thus are connected in Figure 6B. Moreover, the uniqueness value of 0.91 and 0.93 for “protein binding” and “protein-containing complex binding”, respectively, indicated unique terms, and no interaction was observed for these two terms with other enriched terms in molecular function.

### 2.5. Enrichment in Protein Domain for the Protein Targets of Penetratin

The enrichment in the protein families and domain for the protein targets of penetratin was analyzed using the Protein FAMily (PFAM) database. Based on the significant cutoff of *p*-value = 0.05, four enriched terms in PFAM were identified as shown in Table 7. The enriched item “ubiquitin-conjugating enzyme” provided insight into the function of protein targets of penetratin to target destruction of defective protein and synthesis of new proteins. The term “eisosome component PIL1” indicates the targeting of prominent and immobile subcellular structures at the plasma membrane of *Saccharomyces cerevisiae* that functions in lipid homeostasis and stress management for cell wall integrity and cell signaling as well as endocytosis. As endocytosis is the key mechanism for the translocation of penetratin inside the cell, the eisosome component PIL1 can be the key factor. The enriched domain “nucleosome assembly protein” in the protein targets of penetratin pointed toward targeting establishment, maintenance, and dynamics of chromatin of *Saccharomyces cerevisiae*. The “topomyosin like” protein domain indicated the role of the identified penetratin target in cytoskeleton.

### 2.6. Protein-Protein Interaction between the Protein Targets of Penetratin

**Figure 7:**
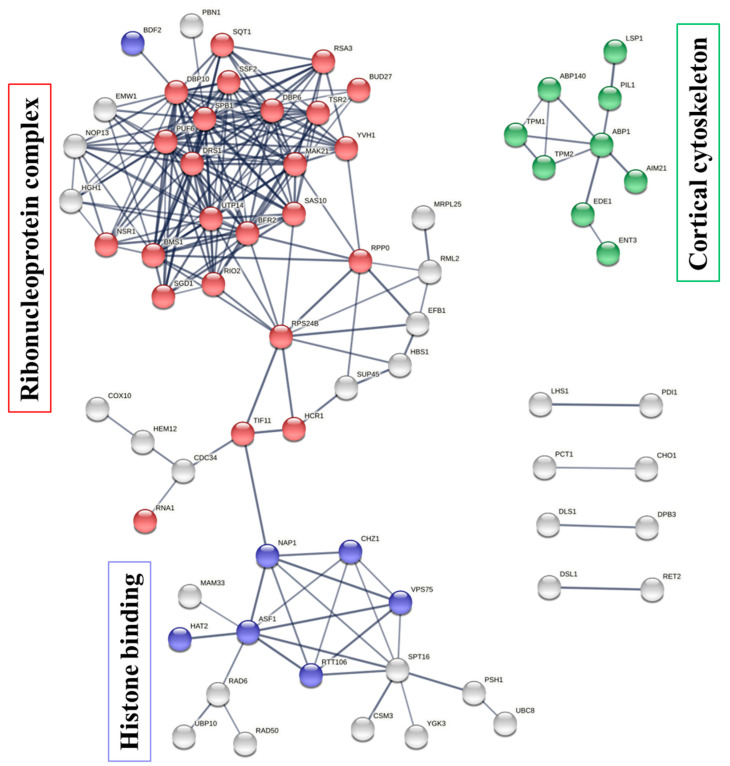
Protein–protein interaction (PPI) networks for the protein targets of penetratin. The protein contributing to ribonucleoprotein complex biogenesis (red color bubbles), histone binding (blue color bubbles), and cortical cytoskeleton (green color bubbles) are shown in three identified PPI networks, respectively. The protein targets of penetratin with white color bubbles in these networks depict functions other than ribonucleoprotein complex biogenesis and histone binding. The thickness of the line connecting these bubbles represents the confidence or strength of PPI.

To illustrate the protein–protein interaction (PPI) network within the protein targets of penetratin, STRING database was used [47]. These networks are important to understanding the functional association of different identified protein targets of penetratin. As illustrated in Figure 7, the protein targets of penetratin show three PPI networks (with PPI enrichment *p*-value of 3.11 × 10^−12^). The three PPI network had maximum number of proteins belong to “ribonucleoprotein complex”, “histone binding”, and “cortical cytoskeleton”, respectively. In the ribonucleoprotein complex network, 25 protein targets of penetratin were involved in ribonucleoprotein complex biogenesis (depicted in red color bubbles). This indicates the protein targets of penetratin are mostly involved in biosynthesis, assembly, and arrangement of ribonucleoprotein complex. Other proteins (represented by white bubbles) in this network indicated proteins with functions other than biogenesis. “Cortical cytoskeleton” is another network enriched in PPI network with 9 protein targets of penetratin (shown in green color bubbles). This indicates that the protein targets of penetratin modulate the shape, size, and motility of *Saccharomyces cerevisiae* and also osmotic stress. Two proteins of eisosome filament (PIL1 and LSP1) were also included in PPI network of cortical cytoskeleton. The third network identified in PPI is “histone binding” and 8 protein targets of penetratin (denoted by blue color bubbles) were involved in this network. The white color bubbles in this network represent the protein targets of penetratin that are involved in functions other than histone binding. The disconnected proteins in PPI network were removed; thus, the number of color bubbles for ribonucleoprotein complex and histone binding were less in Figure 7. These three networks (Figure 7) provided a good summary for all the enriched functions of the 123 protein targets of penetratin, which also represented the major functions of those target proteins in “ribonucleoprotein complex”, “histone binding”, and “cortical cytoskeleton”.

### 2.7. Enrichment in Consensus Motif for the Protein Targets of Penetratin

**Figure 8:**
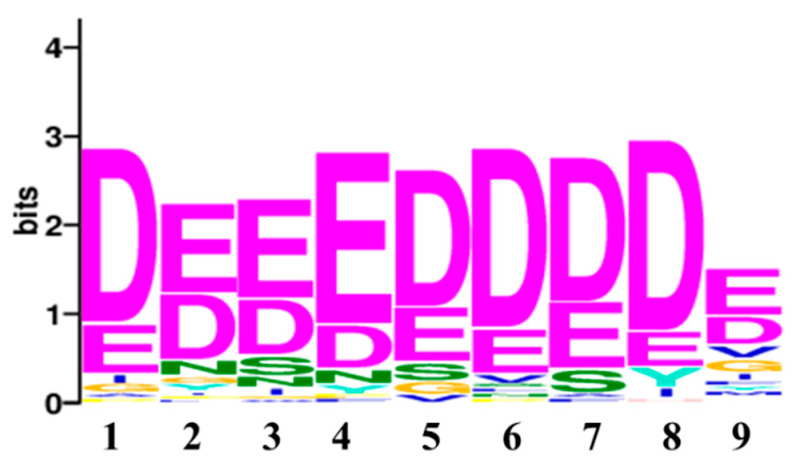
The enriched motif identified in the protein targets of Penetratin, using STREME (Sensitive, Thorough, Rapid, Enriched Motif Elicitation) tool of Multiple Expression motifs for Motif Elicitation (MEME) suite. The identified motif was significantly enriched with a *p*-value of 0.03 and was observed in 57 protein targets of penetratin.

For consensus motif enrichment in the protein targets of penetratin, Sensitive, Thorough, Rapid, Enriched Motif Elicitation (STREME) tool of the Multiple Expression motifs for Motif Elicitation (MEME) suite was employed [48]. The enriched consensus motif of nine amino acids long was identified in the protein targets of penetratin, as illustrated in Figure 8. This enriched motif was significantly enriched with *p*-value = 0.031 and was identified in 57 protein targets of penetratin (i.e., 46.3% of the total protein targets of penetratin).

## 3. Discussion

Penetratin has demonstrated internalization of itself and attached bioactive cargoes molecules to mammalian cells, with minimal cytotoxicity to mammalian cells [49]. Antifungal activity for penetratin is also reported [37,38]. Hence, the use of penetratin as an antifungal agent to target intracellular fungal pathogens without hampering mammalian cells will be highly appreciated. In this study, 123 yeast protein targets of penetratin were screened using *Saccharomyces cerevisiae* proteomic microarrays.

Penetratin is a DNA targeting motif derived from the Antennapedia homeodomain in Drosophila, thus it was obvious to observe enrichment in “transcription, DNA-templated”, “DNA conformation change”, “chromosome organization” and “ribonucleoprotein complex biogenesis” (Table 2) as well as “single-stranded DNA binding” (Table 6) for the protein targets of penetratin.

The internalized penetratin was retrieved from the cytoplasm and nucleus [27]. In accordance, most of the protein targets of penetratin displayed co-localization in cytoplasm and nucleus (Figure 3A, Table 3 and Table 4). Enrichment categories in GO identified several enriched terms. The major enrichment terms for the protein targets of penetratin were depicted by STRING analysis: “ribonucleoprotein complex biogenesis”, “histone binding”, and “cortical cytoskeleton”. The enriched term “cortical cytoskeleton” represents the proteins associated with eisosome filament (i.e., specific filament presents in yeast cell) and actin filament, which are essential components of endocytosis. Endocytosis is reported as a key mechanism in translocation of penetratin inside the cell. The identified protein targets indeed indicates that penetratin targets endocytosis; however, further studies are required to confirmed endocytosis help in translocation only or cause toxicity to yeast cell.

**Figure 9:**
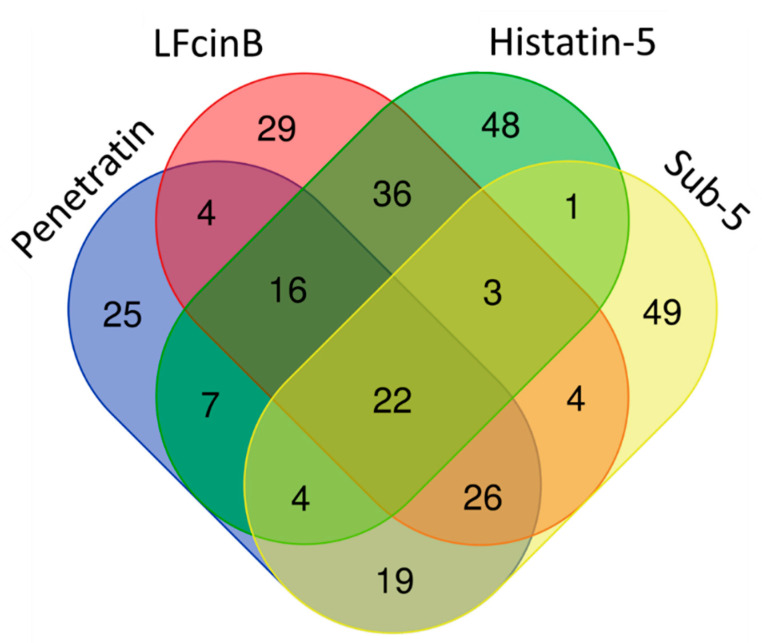
Venn diagram showing the unique and common protein targets of penetratin and three intracellular targeting antifungal AMPs (Lfcin B, Histatin-5, and Sub-5).

CPPs and intracellular targeting AMPs share common features of short cationic peptides and translocation ability across membranes. Both CPPs and AMPs are used as transport vectors for the internalization of desired bioactive cargoes for the diagnosis and treatment of diseases [10]. To understand the differences and similarities between the target pattern of CPP (penetratin) and intracellular targeting AMPs, we compared the protein targets of penetratin with our previously identified protein targets of intracellular targeting antifungal AMPs (LfcinB, Histatin-5 and Sub-5) [40,41]. The comparison results of the protein targets of penetratin and the three antifungal AMPs is displayed in a Venn diagram (Figure 9). As shown in Figure 9, 22 protein targets were the common proteins in all the peptides (penetratin, LfcinB, Histatin-5 and Sub-5) whereas 25, 29, 48 and 49 protein targets were uniquely present only in penetratin, LfcinB, Histatin-5, and Sub-5, respectively. A total of 68, 49 and 71 common protein targets were observed between penetratin and LfcinB, penetratin and Histatin-5, and penetratin and Sub5, respectively.

**Figure 10:**
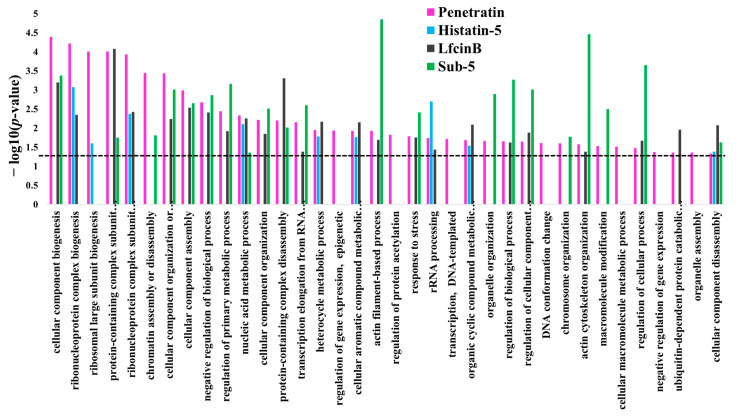
Comparison of the non-redundant enrichment terms in biological process for the protein targets of penetratin with the non-redundant enrichment terms for the protein targets of Histatin-5, Lfcin B, and Sub-5. Enrichment in biological process was individually obtained from DAVID database for the protein targets of penetratin, Histatin-5, LfcinB, and Sub-5. These enrichment results were analyzed by REVIGO to identify non-redundant enrichment terms. The non-redundant enriched terms in the biological process for the protein targets of penetratin, Histatin-5, LfcinB, and Sub-5 were compared. Dotted line represent *p*-value = 0.05.

To observe the similarities and differences in the enrichment pattern of CPP and AMPs, the GO enrichment in biological process was individually analyzed for the protein targets of penetratin, Histatin-5, LfcinB, and Sub-5 using all level categories in DAVID database, followed by the identification of non-redundant enriched items by REVIGO database. The non-redundant enrichment terms for the protein targets of penetratin, Histatin-5, LfcinB, and Sub-5 were compared. The comparison results of non-redundant enrichments items in the GO biological process with their corresponding *p*-values (represented in—log10(*p*-value)) for penetratin, Histatin-5, LfcinB, and Sub-5 is shown in Supplementary Table S3. Figure 10 depicts the comparison results only related to penetratin. With 25 unique protein targets (Figure 9), penetratin display 7 unique enrichment items: “regulation of gene expression, epigenetic”, “regulation of protein acetylation”, “transcription, DNA-templated”, “DNA conformation change”, “cellular macromolecule metabolic process”, “negative regulation of gene expression” and “organelle assembly”, which are mainly DNA-associated biological processes. Two terms, “nucleic acid metabolic process” and “cellular component disassembly”, were commonly enriched in penetratin and the three AMPs. For Histatin-5, Lfcin B and Sub-5 the number of unique enrichment items are 4, 6 and 28, respectively (Supplementary Table S3).

**Figure 11:**
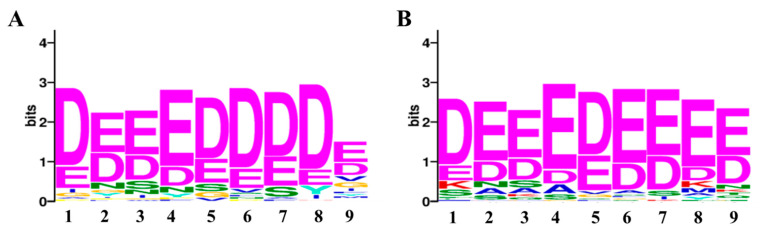
The significantly enriched motif in the yeast protein targets of penetratin (**A**), and Sub-5 (**B**). Using STREME tool, the enriched consensus motif is identified in the protein targets of penetratin and Sub-5. The number of protein targets containing the enriched motif was 46.3% (i.e., 57 proteins) for Penetratin with a *p*-value of 0.031 and 50% (i.e., 64 proteins) for Sub-5 with a *p*-value of 0.019. No significant enriched motif was identified in the protein targets of LfcinB or Histatin-5.

To understand the insights of the binding pattern, enriched consensus motifs in the protein targets of penetratin, Sub-5, LfcinB and Histatin-5 were analyzed using STREME tool (https://meme-suite.org/meme/tools/streme (accessed on 18 November 2021)). The significantly enriched motifs were observed in the protein targets of penetratin (Figure 11A) and Sub-5 (Figure 11B) with significant *p*-value < 0.05. No significantly enriched motif was identified in the protein targets of Lfcin B and Hisitain-5, respectively. The 9 amino acid length consensus motif (DEEEDDDDE) was identified in 57 proteins out of 123 protein targets of penetratin (i.e., 46.3%) with significant *p*-value of 0.031. Similarly, 9 amino acid consensus motif (DEEEDEEEE) was identified in 64 proteins out of 128 protein target of Sub-5 (i.e., 50.0%) with significant *p*-value of 0.019. As identified motifs for penetratin and Sub-5 look similar, we compared the protein targets of penetratin and Sub-5. In total, 71 common protein targets were observed between the total protein targets of penetratin and Sub-5 (Figure 9). Among 57 and 64 proteins that contained the enriched motif for penetratin and Sub-5, we identified 38 common proteins. This result demonstrates ~60% similarity between the enriched motif obtained from the protein targets of penetratin and Sub-5. On the other hand, 19 and 27 unique protein targets of penetratin and Sub-5 containing the consensus motif were identified, which indicate ~40% of uniqueness.

In summary, penetratin is a well-known cell-penetrating peptide studied for the transportation of drugs inside the cell and has also demonstrated to exert antimicrobial activities. In shortage of antifungal agents as well as increasing incidence of drug resistance, penetratin can be the potential drug candidate against intercellular pathogens. In this study, we screened for the potential yeast protein targets of penetratin using *Saccharomyces cerevisiae* proteome microarrays. Protein targets of penetratin showed significant enrichments in terms related to biological process, molecular function and others. Major enrichments were observed in “ribonucleoprotein complex synthesis”, “cortical cytoskeleton” and “histone binding” as depicted by STRING analysis. Cell wall is the unique feature found only in fungi, moreover, endocytosis has been demonstrated as the translocation mechanism of penetratin inside the cells (both yeast and mammal cells). Yeast-specific eisosomes proteins (i.e., PIL1 and LSP1) together with several actin-related proteins identified in the protein targets of penetratin are the critical components of endocytosis. Further studies are required to confirm penetratin targeting to endocytosis aid in translocation or cause toxicity to yeast cell. We further compared the protein targets of penetratin with our previously identified protein targets for intracellular antifungal AMPs (LfcinB, Histain-5 and Sub-5) in terms of proteins, enriched terms in biological process, and motif enrichment to understand the similarities and differences between CPP and intracellular targeting AMPs. As CPPs and AMPs share cationic properties, we did observe several similarities in terms of common protein targets and enrichment between penetratin and antifungal AMPs (LfcinB, Histatin-5 and Sub-5). Moreover, several unique protein targets and enrichment terms, demonstrated their unique features. Hence, the protein targets of penetratin identified in this study not only provided the insight to understand the antifungal activity of penetratin but also demonstrated their unique target pattern, which might be beneficial for designing a novel antifungal therapeutics agent against intracellular fungal pathogen.

## 4. Materials and Methods

### 4.1. Expression and Purification of Entire Yeast Proteome

*Saccharomyces cerevisiae* proteome library [50] consists of ~5800 individual clones with open reading frames of the individual protein in *Saccharomyces cerevisiae*. To obtain the entire proteins of *Saccharomyces cerevisiae*, these clones were cultured and the proteins were expressed and purified individually following the previous protocol [41,51,52,53]. Methods for the expression of proteins using 96-plate format are briefly described below. Firstly, *Saccharomyces cerevisiae* (i.e., yeast) clones stored at –80 °C were thawed and inoculated on a synthetic complete medium containing glucose but lacking URA3 (SC-URA3-glucose) agar plates. After obtaining 2 mm diameter of colonies by incubation for 48 h at 30 °C, they were transferred to liquid SC-URA3-glucose medium in 96-deep well plates and incubated with shaking for 24 h at 30 °C. These clones were transferred to SC-URA3-raffinose medium and incubated with shaking for ~16 h at 30 °C. At optical density at 600 nm (OD_600_) of yeast clones = 0.6–1.0, 2% galactose were added to induce protein. These clones were incubated with shaking for 4 h at 30 °C. Yeast cell pellets were collected by centrifugation and temporarily stored at −20 °C.

For protein purification, yeast cell pellets were ruptured with zirconia beads (BioSpec Products Inc. Bartlesville, OK, USA) in freshly prepared lysis buffer [1 µM MG132, 1 mM PMSF, 50 µM calpain Inhibitor I (LLnL), and 50× dilution of Roche protease inhibitor] incubating on microplate vortex for 30 min at 4 °C. 96-well plates were centrifuged and supernatants were transferred to new plates followed by the addition of 100 µL pre-washed glutathione (GSH) Sepharose 4B beads (GE Healthcare, Chicago, IL, USA). The 96-well plates were sealed and vertically placed on an incubator with shaking (80 rpm) for 80 min at 4 °C. Using wide bore tips, mixtures from 96-well plates were transferred to 96-well filter plates. Firstly, the mixtures were washed by wash buffer I (50 mM Tris-base, 500 mM NaCl, 1 mM EDTA, 10% glycerol, 0.1% TritonX-100, 0.1% 2-mercaptoethanol, and 1 mM PMSF) for 6 times followed by wash buffer II (50 mM HEPES, 100 mM NaCl, 1 mM EDTA, 10% glycerol, 0.1% 2-mercaptoethanol, and 1 mM PMSF) for 6 times. Secondly, the bottom of 96-well filter plates was sealed and 50 µL elution buffer (50 mM HEPES, 100 mM NaCl, 10% glycerol, 0.03% TritonX-100, and 40 mM reduced glutathione) was added. The 96-well filter plates were incubated with vigorous shaking for 1 h at 4 °C. Finally, the purified proteins were obtained by centrifugation of 96-well filter plates at 4000 rpm for 2 min. Most of the materials used in this experiment were purchased from Sigma Aldrich (Sigma Aldrich St. Louis, MO, USA).

### 4.2. Fabrication of Yeast Proteome Microarrays

The purified yeast proteins (~5800) were used for the fabrication of yeast proteome microarrays following the previous protocol [40,41]. Briefly, purified yeast proteins in 96-well plates were transferred to 384-well plates. Using 48 microarray pins equipped CapitalBio SmartArrayer 136—a high-throughput plate-ready microarray spotter (Capitalbio Corporation, Beijing, China), duplicate protein spots of ~5800 yeast proteins and landmark proteins were spotted on aldehyde-coated glass slides. Fabrication of yeast proteome microarrays was performed in 4 °C with controlled humidity (below 40%) and microarray pins were washed after each duplicate spot. Fabricated yeast proteome microarrays (entire yeast proteome spotted on aldehyde-coated glass slides) were immobilized by incubating overnight at 4 °C. Later, these yeast proteome microarrays were placed in slide boxes, vacuum-sealed, and stored at −80 °C. Yeast proteome microarrays were probed with anti-His antibody-DyLight 550 (Rockland Immunochemicals Inc., Pottstown, PA, USA) and scanned using LuxScan^TM^ (10 K Microarray Scanner; CapitalBio Inc., Beijing, China) to determine the shape, size, and uniformity for each protein on yeast proteome microarray.

### 4.3. Yeast Proteome Microarray Assays of Penetratin

Penetratin with N-terminal biotin (RQIKIWFQNRRMKWKK—Biotin) was purchased from Kelowna International Scientific Inc. (Taipei, Taiwan). For yeast proteome microarrays assays of penetratin, yeast proteome microarrays (stored at −80 °C) were submerged in PBS-T (PBS buffer with 0.05% Tween 20) and incubated with shaking (50 rpm) at room temperature (RT) for 2 min. Yeast proteome microarrays were blocked with blocking buffer (3% BSA in PBS) at RT for 1 h with gentle agitation (50 rpm). Yeast proteome microarrays were washed with PBS-T with shaking at RT for 5 min (one time). Biotinylated penetratin was probed on yeast proteome microarrays and incubation with shaking (50 rpm) at RT for 1 h. The yeast proteome microarrays were washed with PBS-T by incubating with shaking at RT for 5 min (one time). Further, yeast proteome microarrays were probed with streptavidin-DyLight 650 and anti-His antibody-DyLight™ 550, respectively and incubated with shaking (50 rpm) at RT for 1 h. Yeast proteome microarrays were washed with PBS-T by incubating with shaking at RT for 5 min (three time). Yeast proteome microarrays were dried by centrifugation at 1000 rpm for 1 min and later scanned with LuxScan to detect the binding partners of penetratin and His-tag yeast proteins.

### 4.4. Analysis of Protein Targets of Penetratin

Scanned yeast proteome microarrays (a.k.a yeast chips) probed with penetratin were opened using GenePix Pro 6.0 software (Axon Instruments, Union City, CA, USA). Each protein spot on the yeast proteome microarrays was aligned with their respective name and the binding signal of penetratin and anti-His tag was exported as GPR files (in triplicate). GRP files were opened with excel and data were analyzed together using below-mentioned statistical criteria. The signal intensity of each protein spot on proteome microarrays was normalized for penetratin and anti-His-tag using median scaling normalization. Cutoff parameter of signal intensity greater than 100 was applied to each spot on the yeast proteome microarray for penetratin. Coefficient variation (CV) lower than 0.5 was applied between duplicate protein spots on a single yeast proteome microarray. The ratio cutoff between penetratin and anti-His signal of each protein spot was greater than 0.5. Lastly, the signal intensity of penetratin greater than the local cutoff of two standard deviation (SD) above the signal means for each spot was used to select the protein targets of penetratin from triplicate yeast proteome microarrays assay. Each protein spot (in duplicate) from the triplicate yeast chips of the identified protein targets of penetratin was confirmed to have outstanding signals than the background. The final list of protein targets of penetratin that satisfied all the above criteria was obtained.

### 4.5. Bioinformatics Analysis of Protein Targets of Penetratin

Name, function and subcellular location of 123 identified yeast protein targets of penetratin were obtained using UniProt (Universal Protein Resource) database (https://www.uniprot.org/) on 16 September 2021 [42]. The yeast protein targets of penetratin were subjected to different bioinformatics tools to gain significant understanding of the role/effect of these proteins in yeast.

#### 4.5.1. Functional Classification

The identified protein targets of penetratin were classified using PANTHER (Protein ANalysis THrough Evolutionary Relationships) classification system version 16.0 [43]. Protein ID of all the protein targets of penetratin were entered on the online platform of PANTHER (http://www.pantherdb.org/ (accessed on 19 September 2021)) and “*Saccharomyces cerevisiae*” was selected as “select organism”, keeping all other parameters as default.

#### 4.5.2. Enrichment Identification

To identify the enrichment in the protein targets of penetratin, DAVID database (Database for Annotation, Visualization, and Integrated Discovery) version 6.8 (https://david.ncifcrf.gov/home.jsp) [44,45] was accessed on 16 August 2021. Protein ID of protein targets of penetratin was entered on the online platform of DAVID, “EnSEMBL_GENE_ID” was selected as select identifier, “Gene List” was selected as List Type and “*Saccharomyces cerevisiae* S288C” was selected as background. Functional annotation tool was chosen to obtain the enrichment in protein targets of penetratin. Significantly enriched categories were selected using *p*-value < 0.05. Based on stringent *p*-value cutoff < 0.05, significant enrichments were obtained in gene ontology (for biological process, cellular component and molecular function in all levels) and protein families (PFAM) domain.

#### 4.5.3. Identification of Non-Redundant Enrichment Terms

Based on pseudo-hierarchy or directed acyclic graph (DAG) structure of Gene Ontology Consortium, DAVID provides annotate lists of genes at different levels within the DAG. To acquire all the complete enrichment list for the protein targets of penetratin, Gene Ontology enrichment in all level was selected in DAVID. These enrichment lists contain several redundant enrichment terms. REVIGO database (REduce + VIsualize Gene Ontology) (http://revigo.irb.hr/) [46] was used on 26 September 2021 to summarize the long list of GO enrichment terms by removing the redundant GO terms. Parameters used for REVIGO analysis were as follows: the resulting list to “Small (0.5)”, *p*-values associated with GO terms represent: “Lower value is better” and species selected as *Saccharomyces cerevisiae*. Those enrichment terms that were not unique and shared large similarities with other terms were removed by REVIGO. “Frequency” represents the percentage of *Saccharomyces cerevisiae* proteins in UniProt for the selected GO terms in the gene ontology Annotation (GOA) database ((http://www.ebi.ac.uk/goa) accessed on 16 November 2021). The more general GO term is denoted by the higher percentage of frequency. “Uniqueness” and “dispensability” values provided by REVIGO are the semantic distance of the selected term to the other terms. Uniqueness indicates if the selected term is an outlier compared to the entire list. Dispensability on the other hand compares the selected term with other semantic close terms.

#### 4.5.4. Protein–Protein Interaction

To obtain the connection between the protein targets of penetratin in terms of physical interactions as well as functional associations, STRING database version 11.5 (https://string-db.org/) [47] was accessed on 27 October 2021. Parameter used for protein–protein interaction between the protein targets of penetratin on STRING database were: search for “multiple proteins”, organism selected “*Saccharomyces cerevisiae*”, minimum required interaction score was “high confidence (0.700)” and network display options include “hide disconnected nodes in the network”.

#### 4.5.5. Motif Search

The protein targets of penetratin were analyzed for ungapped enriched motif using STREME version 5.4.1 (Sensitive, Thorough, Rapid, Enriched Motif Elicitation) (https://meme-suite.org/meme/tools/streme) [48] on 26 September 2021. The Fasta files of protein targets of penetratin were upload to STREME tools to analyze enriched motif using default parameters.

#### 4.5.6. Venn Diagram

To categorize the common and unique protein targets between Penetratin, LfcinB, Histatin-5 and Sub-5, a Venn diagram was generated using online platform (http://bioinformatics.psb.ugent.be/webtools/Venn/ (accessed on 27 September 2021)).

## Figures and Tables

**Table 1:** Enriched top 20 terms in biological process for the protein targets of penetratin.

Term	Enrichment Items in Biological Process	*p*-Value	Hit in This Category	Total Gene in This Category
GO:0044085	cellular component biogenesis	0.000041	43	1230
GO:0022613	ribonucleoprotein complex biogenesis	0.000061	24	502
GO:0042254	ribosome biogenesis	0.000074	21	406
GO:0042273	ribosomal large subunit biogenesis	0.000098	11	121
GO:0043933	macromolecular complex subunit organization	0.000099	35	938
GO:0000027	ribosomal large subunit assembly	0.000115	7	41
GO:0071826	ribonucleoprotein complex subunit organization	0.000117	14	202
GO:0022618	ribonucleoprotein complex assembly	0.000259	13	190
GO:0006333	chromatin assembly or disassembly	0.000356	8	70
GO:0071840	cellular component organization or biogenesis	0.000368	69	2611
GO:0042255	ribosome assembly	0.000748	8	79
GO:0006334	nucleosome assembly	0.001026	5	24
GO:0022607	cellular component assembly	0.001034	30	848
GO:0031497	chromatin assembly	0.001163	6	42
GO:0061572	actin filament bundle organization	0.001621	5	27
GO:0051017	actin filament bundle assembly	0.001621	5	27
GO:0048523	negative regulation of cellular process	0.001693	23	592
GO:0031324	negative regulation of cellular metabolic process	0.001968	19	447
GO:0048519	negative regulation of biological process	0.002100	23	602

**Table 2:** Non-redundant enrich items in the biological process of gene ontology provided by REVIGO analysis.

Term	Enrichment Items in Biological Process	*p*-Value	Frequency	Uniqueness	Dispensability
GO:0044085	cellular component biogenesis	0.000041	19.1116	0.8228	0.2438
GO:0022613	ribonucleoprotein complex biogenesis	0.000061	7.7905	0.7860	0.4369
GO:0042273	ribosomal large subunit biogenesis	0.000098	1.9891	0.7819	0.0000
GO:0043933	protein-containing complex subunit organization	0.000099	10.0448	0.8178	0.3204
GO:0071826	ribonucleoprotein complex subunit organization	0.000117	2.8510	0.8028	0.1694
GO:0071840	cellular component organization or biogenesis	0.000368	36.7479	0.9841	0.0140
GO:0048519	negative regulation of biological process	0.002100	12.1167	0.7144	0.4198
GO:0090304	nucleic acid metabolic process	0.004643	21.1835	0.8481	0.4444
GO:0016043	cellular component organization	0.006102	31.9907	0.8043	0.4532
GO:0046483	heterocycle metabolic process	0.011293	26.0733	0.9347	0.1199
GO:0040029	regulation of gene expression, epigenetic	0.011568	1.7073	0.7056	0.0000
GO:0006725	cellular aromatic compound metabolic process	0.011773	25.9075	0.9348	0.2032
GO:0030029	actin filament-based process	0.011778	2.0554	0.9908	0.0076
GO:1901983	regulation of protein acetylation	0.014944	0.1823	0.7351	0.2597
GO:0006950	response to stress	0.016292	12.4979	1.0000	0.0000
GO:0006351	transcription, DNA-templated	0.019146	3.9781	0.8306	0.0083
GO:1901360	organic cyclic compound metabolic process	0.020540	26.9683	0.9414	0.1791
GO:0006996	organelle organization	0.021870	23.0068	0.7892	0.4854
GO:0050789	regulation of biological process	0.022090	28.7419	0.7210	0.3022
GO:0044087	regulation of cellular component biogenesis	0.022672	3.2819	0.7643	0.2373
GO:0071103	DNA conformation change	0.024730	2.9007	0.7582	0.2184
GO:0051276	chromosome organization	0.025070	9.1994	0.7828	0.3818
GO:0030036	actin cytoskeleton organization	0.026612	1.9891	0.7952	0.3034
GO:0043412	macromolecule modification	0.029397	13.9234	0.9157	0.2066
GO:0044260	cellular macromolecule metabolic process	0.030521	32.8858	0.8886	0.3481
GO:0010629	negative regulation of gene expression	0.042087	3.9450	0.5963	0.4389
GO:0006511	ubiquitin-dependent protein catabolic process	0.043615	3.9450	0.9057	0.2312
GO:0070925	organelle assembly	0.043644	2.8013	0.7623	0.3573
GO:0022411	cellular component disassembly	0.046521	2.2543	0.8537	0.2109

**Table 3:** Enriched items in cellular component of gene ontology for the protein targets of penetratin.

Term	Enrichment Items in Cellular Component	*p*-Value	Hit in This Category	Total Gene in This Category
GO:0043232	intracellular non-membrane-bounded organelle	0.00000244	52	1429
GO:0043228	non-membrane-bounded organelle	0.00000244	52	1429
GO:0030684	preribosome	0.00000437	16	191
GO:0030687	preribosome, large subunit precursor	0.0000104	11	91
GO:0030863	cortical cytoskeleton	0.000103	9	75
GO:0005730	nucleolus	0.000187	17	293
GO:0031981	nuclear lumen	0.000215	33	863
GO:0032991	macromolecular complex	0.000681	63	2256
GO:0044428	nuclear part	0.000781	41	1260
GO:0070013	intracellular organelle lumen	0.000828	38	1136
GO:0043233	organelle lumen	0.000828	38	1136
GO:0030529	intracellular ribonucleoprotein complex	0.001374	28	756
GO:1990904	ribonucleoprotein complex	0.001374	28	756
GO:0031974	membrane-enclosed lumen	0.001374	39	1209
GO:0005634	nucleus	0.002179	63	2347
GO:0030864	cortical actin cytoskeleton	0.003055	7	73
GO:0000781	chromosome, telomeric region	0.005892	8	110
GO:0015629	actin cytoskeleton	0.006122	7	84
GO:0044448	cell cortex part	0.006571	9	141
GO:0005938	cell cortex	0.007552	10	175
GO:0099568	cytoplasmic region	0.007552	10	175
GO:0032432	actin filament bundle	0.007643	3	7
GO:0005654	nucleoplasm	0.012738	15	366
GO:0098687	chromosomal region	0.021542	10	208
GO:0005694	chromosome	0.022792	17	472
GO:0044427	chromosomal part	0.022923	16	433
GO:0030479	actin cortical patch	0.030382	5	60
GO:0044446	intracellular organelle part	0.03059	72	3060
GO:0044422	organelle part	0.031893	72	3065
GO:0061645	endocytic patch	0.032033	5	61
GO:0036286	eisosome filament	0.039195	2	2

**Table 4:** Non-redundant enrich items in cellular component of gene ontology analyzed by REVIGO.

Term	Enrichment Items in Cellular Component	*p*-Value	Frequency	Uniqueness	Dispensability
GO:0043228	non-membrane-bounded organelle	0.00000244	23.1776	0.9664	0.0243
GO:0030684	preribosome	0.00000437	2.7833	0.9287	0.0000
GO:0030863	cortical cytoskeleton	0.000103	1.2094	0.5535	0.4336
GO:0005730	nucleolus	0.000187	4.8708	0.5807	0.0000
GO:0032991	protein-containing complex	0.000681	35.5533	1.0000	0.0000
GO:1990904	ribonucleoprotein complex	0.001374	9.1286	0.9553	0.2231
GO:0031974	membrane-enclosed lumen	0.001374	17.3459	0.9958	0.0025
GO:0005634	nucleus	0.002179	39.6620	0.9571	0.0456
GO:0099568	cytoplasmic region	0.007552	0.2154	0.9889	0.0037
GO:0005938	cell cortex	0.007552	3.0649	0.8336	0.0165
GO:0098687	chromosomal region	0.021542	3.2638	0.7299	0.3486

**Table 5:** Enrichment items in molecular function of gene ontology for protein targets of penetratin.

Term	Enrichment Items in Molecular Function	*p*-Value	Hit in This Category	Total Gene in This Category
GO:0042393	histone binding	0.00000216	9	46
GO:0005515	protein binding	0.00000439	36	854
GO:0032403	protein complex binding	0.006213	7	86
GO:0051015	actin filament binding	0.00639	4	20
GO:0044877	macromolecular complex binding	0.009991	12	255
GO:0008135	translation factor activity, RNA binding	0.028574	5	60
GO:0003697	single-stranded DNA binding	0.031749	5	62
GO:0003786	actin lateral binding	0.03852	2	2
GO:0061630	ubiquitin protein ligase activity	0.039322	4	39
GO:0061659	ubiquitin-like protein ligase activity	0.039322	4	39

**Table 6:** Non-redundant enrich items in molecular function of gene ontology identified by REVIGO analysis.

Term	Enrichment Items in Molecular Function	*p*-Value	Frequency	Uniqueness	Dispensability
GO:0042393	histone binding	0.00000216	0.8952	0.9099	0.0000
GO:0005515	protein binding	0.00000439	40.9151	0.9106	0.0815
GO:0044877	protein-containing complex binding	0.006213	3.3985	0.9306	0.0431
GO:0051015	actin filament binding	0.006390	0.5139	0.7713	0.1057
GO:0008135	translation factor activity, RNA binding	0.028574	0.8952	0.8246	0.0431
GO:0003697	single-stranded DNA binding	0.031749	1.1107	0.8838	0.2688
GO:0061630	ubiquitin protein ligase activity	0.039322	0.9284	0.7436	0.2621

**Table 7:** Enrichment categories in protein domain analyzed by PFAM for the protein targets of Sub5.

Term	Enrichment in PFAM	*p*-Value	Hit in This Category	Total Gene in This Category	Genes
PF00179	Ubiquitin-conjugating enzyme	0.038	3	14	YDR054C, YEL012W, YGL058W
PF13805	Eisosome component PIL1	0.044	2	2	YGR086C, YPL004C
PF00956	Nucleosome assembly protein (NAP)	0.044	2	2	YNL246W, YKR048C
PF12718	Tropomyosin like	0.044	2	2	YNL079C, YIL138C

## Data Availability

Not applicable.

## Supplementary Materials

The following are available online at https://www.mdpi.com/article/10.3390/ijms23020712/s1.
