# Supplementary material for: Systematic Screening of Penetratin’s Protein Targets by Yeast Proteome Microarrays
Source: Int J Mol Sci. 2022 Jan 10;23(2):712. doi: 10.3390/ijms23020712 (PMC8775591; doi:10.3390/ijms23020712)
Supplement: Supplementary file 1 [file ijms-23-00712-s001.zip › ijms-1497189-supplementary.pdf]

Supplementary Materials

Systematic Screening of Penetratin's Protein Targets by Yeast Proteome Microarrays

Pramod Shah [1,2] and Chien-Sheng Chen [1,3,*]

[1] Institute of Systems Biology and Bioinformatics, Department of Biomedical Sciences and Engineering, College of Health Sciences and Technology, National Central University, Jhongli 300, Taiwan; prokonp@gmail.com
[2] Department of Nutritional Science, Fu Jen Catholic University, New Taipei City 242, Taiwan
[3] Department of Food Safety/Hygiene and Risk Management, College of Medicine, National Cheng Kung University, Tainan 701, Taiwan
* Correspondence: cchen103@gmail.com; Tel.: +886-6-235-3535 (ext. 5964); Fax: +886-6-275-2484

Table S1. The list of 123 identified protein targets of penetration from the triplicate yeast proteome microarrays.

Protein ID	Uniprot ID	Protein names	subcellular location
YNL245C	P53854	Pre-mRNA-splicing factor CWC25	Nucleus, Spliceosome
YJL115W	P32447	Histone chaperone ASF1	Nucleus
YGL090W	P53150	Ligase-interacting factor 1	Cytoplasm, Nucleus
YDL153C	Q12136	Something about silencing protein 10	nucleolus
YMR235C	P11745	Ran GTPase-activating protein 1	Cytoplasm
YGL242C	P53066	Ankyrin repeat-containing protein YGL242C	unknown
YML062C	P33441	THO complex subunit MFT1	Nucleus
YEL012W	P28263	Ubiquitin-conjugating enzyme E2-24 kDa	Cytoplasm
YGR086C	P53252	Sphingolipid long chain base-responsive protein PIL1	Lipid droplet
YOL054W	Q12161	RING finger protein PSH1	Nucleus
YKL160W	P36053	Transcription elongation factor 1	Nucleus
YGL207W	P32558	FACT complex subunit SPT16	Chromosome, Nucleus
YDR054C	P14682	Ubiquitin-conjugating enzyme E2-34 kDa	Cytoplasm, Nucleus
YGL208W	P34164	SNF1 protein kinase subunit beta-2	Cell membrane, Cytoplasm, Membrane
YPL190C	P38996	Nuclear polyadenylated RNA-binding protein 3	Nucleus, nucleoplasm
YOR054C	Q08438	Phosphopantothenoylcysteine decarboxylase subunit VHS3	Cytoplasm
YNL250W	P12753	DNA repair protein RAD50	Mitochondrion, Nucleus
YLR114C	Q12500	Late secretory pathway protein AVL9	Cytoplasm
YDR346C	Q05515	Survival factor 1	Cytoplasm, Nucleus
YMR260C	P38912	Eukaryotic translation initiation factor 1A	cytoplasmic
YCR088W	P15891	Actin-binding protein	Cytoplasm, Cytoskeleton
YLR055C	P38915	Transcription factor SPT8	Nucleus
YIL104C	P40486	Protein SHQ1	Nucleus
YOR239W	Q08641	tRNA(Thr) (cytosine(32)-N(3))-methyltransferase	Cytoplasm, Cytoskeleton
YGL058W	P06104	Ubiquitin-conjugating enzyme E2 2	Cytoplasm, Nucleus
YCL054W	P25582	27S pre-rRNA (guanosine(2922)-2'-O)-methyltransferase	nucleolus
YPL004C	Q12230	Sphingolipid long chain base-responsive protein LSP1	Cytoplasm
YNL207W	P40160	Serine/threonine-protein kinase RIO2	Cytoplasm, Nucleus

YLL008W	P32892	ATP-dependent RNA helicase DRS1	nucleolus
YGR159C	P27476	Nuclear localization sequence-binding protein	nucleolus
YGR187C	P48362	Protein HGH1	Nucleus, Cytoplasm
YML093W	Q04500	U3 small nucleolar RNA-associated protein 14	nucleolus
YHR156C	P38852	Protein LIN1	Nucleus
YFL034W	P43564	Probable lipase MIL1	Golgi apparatus, Endosome
YJL123C	P47018	Maintenance of telomere capping protein 1	Cytoplasm, Cytoplasmic vesicle
YNR038W	P53734	ATP-dependent RNA helicase DBP6	nucleolus
YKL028W	P36100	Transcription initiation factor IIE subunit alpha	Nucleus
YKR048C	P25293	Nucleosome assembly protein	Cytoplasm, Nucleus
YLR331C	O13555	Uncharacterized protein JIP3	unknown
YMR091C	P32832	Chromatin structure-remodeling complex subunit RSC7	Nucleus
YNL186W	P53874	Ubiquitin carboxyl-terminal hydrolase 10	Chromosome, Nucleus, Telomere
YDR060W	Q12176	Ribosome biogenesis protein MAK21	nucleolus
YJR072C	P47122	GPN-loop GTPase 1	Cytoplasm
YPR152C	Q06525	Pre-mRNA-splicing factor URN1	Nucleus, Spliceosome
YBL011W	P32784	Glycerol-3-phosphate O-acyltransferase 1	Endoplasmic reticulum membrane
YBL047C	P34216	EH domain-containing and endocytosis protein 1	Cytoplasm
YNL099C	P50946	Putative tyrosine-protein phosphatase OCA1	Cytoplasm
YDR496C	Q04373	Pumilio homology domain family member 6	nucleolus
YGL256W	P10127	Alcohol dehydrogenase 4	Mitochondrion
YIL138C	P40414	Tropomyosin-2	Cytoplasm, Cytoskeleton
YBR296C	P38361	Phosphate permease PHO89	other
YPL217C	Q08965	Ribosome biogenesis protein BMS1	Cytoplasm, Nucleus
YNL254C	P53850	Restriction of telomere capping protein 4	Cytoplasm, Nucleus
YLR435W	Q06672	Pre-rRNA-processing protein TSR2	Cytoplasm, Nucleus
YIR012W	P35184	Ribosome assembly protein SQT1	Cytosol
YGR202C	P13259	Choline-phosphate cytidylyltransferase	other
YFR015C	P23337	Glycogen [starch] synthase isoform 1	Mitochondrion, cytoplasm
YJL170C	P46993	Protein ASG7	Endomembrane system
YNL079C	P17536	Tropomyosin-1	Cytoplasm, Cytoskeleton
YKL195W	P36046	Mitochondrial intermembrane space import and assembly protein 40	Mitochondrion inner membrane
YFR051C	P43621	Coatomer subunit delta	Cytoplasm, Cytoplasmic vesicle, Golgi apparatus, Membrane
YLR340W	P05317	60S acidic ribosomal protein P0	Cytoplasm
YAL003W	P32471	Elongation factor 1-beta	Cytosol
YNL258C	P53847	Protein transport protein DSL1	Endoplasmic reticulum, Membrane
YLR192C	Q05775	Eukaryotic translation initiation factor 3 subunit J	Cytoplasm
YDL031W	Q12389	ATP-dependent RNA helicase DBP10	nucleolus
YGL209W	P53035	Regulatory protein MIG2	Nucleus
YIR003W	P40563	Altered inheritance of mitochondria protein 21	Cytoplasm, Cytoskeleton
YDL070W	Q07442	Bromodomain-containing factor 2	Cytoplasm, Nucleus
YKL073W	P36016	Heat shock protein 70 homolog LHS1	Endoplasmic reticulum
YMR025W	Q04368	Cop9 signalosome-interactor 1	Cytoplasm, Nucleus, Signalosome
YKL091C	P33324	CRAL-TRIO domain-containing protein YKL091C	Nucleus
YLR295C	Q12349	ATP synthase subunit H, mitochondrial	Mitochondrion
YEL056W	P39984	Histone acetyltransferase type B subunit 2	Cytoplasm, Nucleus
YDR273W	Q05610	Donuts protein 1	Other

YFL023W	P43573	Bud site selection protein 27	Cytoplasm
YIL070C	P40513	Mitochondrial acidic protein MAM33	Mitochondrion
YER030W	P40019	Histone H2A.Z-specific chaperone CHZ1	Nucleus
YDR299W	Q06631	Protein BFR2	nucleolus
YBR143C	P12385	Eukaryotic peptide chain release factor subunit 1	Cytoplasm
YKR084C	P32769	Elongation factor 1 alpha-like protein	Cytoplasm
YER116C	P40072	E3 ubiquitin-protein ligase complex SLX5-SLX8 subunit SLX8	Centromere, Chromosome, Kinetochore, Nucleus
YGR126W	P53274	Uncharacterized protein YGR126W	Nucleus, cytoplasm
YNL217W	P40152	Putative metallophosphoesterase YNL217W	Vacuole
YJL065C	P40366	Protein DLS1	Nucleus
YIL154C	P32351	Sugar utilization regulatory protein IMP2	cytoplasm
YGR076C	P23369	54S ribosomal protein L25, mitochondrial	Mitochondrion
YIL069C	P0CX32	40S ribosomal protein S24-B	Cytoplasm
YFR040W	P43612	SIT4-associating protein SAP155	Cytoplasm
YLR221C	Q05942	Ribosome assembly protein 3	nucleolus
YNL206C	P40161	Histone chaperone RTT106	Chromosome, Nucleus
YDR312W	Q12153	Ribosome biogenesis protein SSF2	nucleolus
YNL246W	P53853	Vacuolar protein sorting-associated protein 75	Nucleus
YGL130W	Q01159	mRNA-capping enzyme subunit alpha	Nucleus
YNL175C	P53883	Nucleolar protein 13	nucleolus
YIR026C	Q02256	Tyrosine-protein phosphatase YVH1	Nucleus, cytoplasm
YKL005C	P36106	Transcription factor BYE1	Nucleus
YLL048C	P32386	ATP-dependent bile acid permease	Vacuole membrane
YBR278W	P27344	DNA polymerase epsilon subunit C	Nucleus
YOL128C	Q12222	Glycogen synthase kinase-3 homolog ...	Nucleus
YER119C	P40074	Vacuolar amino acid transporter 6	Vacuole membrane
YNL313C	P42842	Essential for maintenance of the cell wall protein 1	Cytoplasm, Nucleus
YDR068W	P54858	Protein DOS2	Cytoplasm
YMR173W	P18899	Stress protein DDR48	Cytosol
YLR336C	Q06132	Suppressor of glycerol defect protein 1	nucleolus
YCL043C	P17967	Protein disulfide-isomerase	Endoplasmic reticulum lumen
YLR161W	P0CE98	Putative uncharacterized protein YLR161W	unknown
YGL017W	P16639	Arginyl-tRNA--protein transferase 1	Cytoplasm
YDR047W	P32347	Uroporphyrinogen decarboxylase	Cytoplasm, Nucleus
YBR092C	P24031	Constitutive acid phosphatase	Cell Wall, Endoplasmic reticulum
YGL134W	P53124	PHO85 cyclin-10	Cytoplasm
YJR125C	P47160	Epsin-3	Cytoplasm, Cytoplasmic vesicle, Golgi apparatus, Membrane
YHR158C	P38853	Kelch repeat-containing protein 1	cell cortex, cytoplasm, prospore membrane
YDL173W	Q12515	Protein PAR32	Cytoplasm
YPL172C	P21592	Protoheme IX farnesyltransferase, mitochondrial	Mitochondrion membrane
YDR525W	Q04413	Uncharacterized protein API2	unknown
YEL050C	P32611	54S ribosomal protein RML2, mitocho...	Mitochondrion
YER026C	P08456	CDP-diacylglycerol--serine O-phosphatidyltransferase	Mitochondrion outer membrane, Endoplasmic reticulum membrane
YMR048W	Q04659	Chromosome segregation in meiosis protein 3	Nucleus
YER096W	P39000	Protein SHC1	Cytoplasm, Cytoplasmic granule Membrane
YCL052C	P25580	Protein PBN1	Endoplasmic reticulum membrane

YDR065W	Q12167	Required for respiratory growth protein 1, mitochondrial	Mitochondrion
YGR097W	P48361	Activator of SKN7 protein 10	Cytoplasm

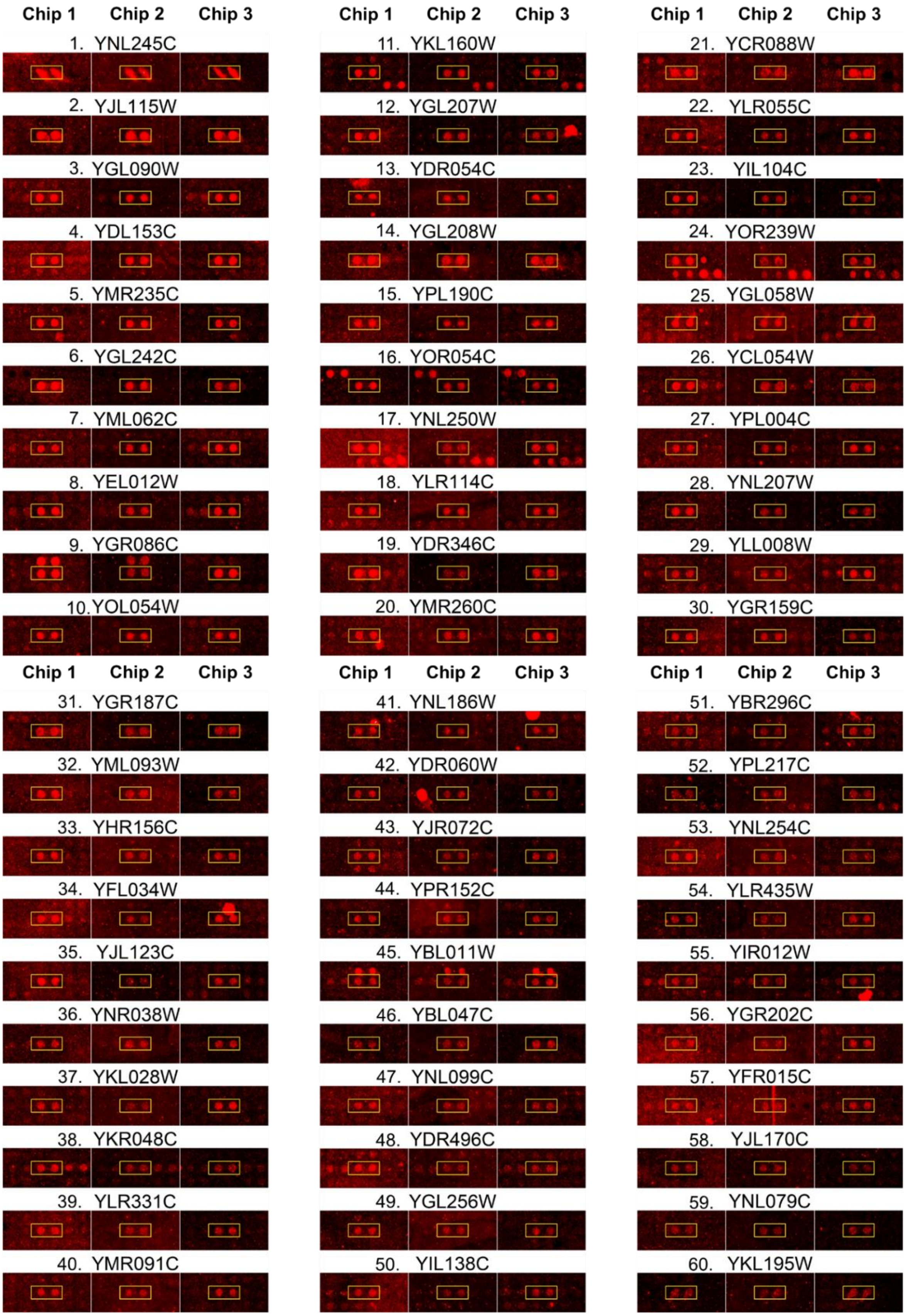
Chip 1 Chip 2 Chip 3
1. YNL245C
2. YJL115W
3. YGL090W
4. YDL153C
5. YMR235C
6. YGL242C
7. YML062C
8. YEL012W
9. YGR086C
10. YOL054W
11. YKL160W
12. YGL207W
13. YDR054C
14. YGL208W
15. YPL190C
16. YOR054C
17. YNL250W
18. YLR114C
19. YDR346C
20. YMR260C
21. YCR088W
22. YLR055C
23. YIL104C
24. YOR239W
25. YGL058W
26. YCL054W
27. YPL004C
28. YNL207W
29. YLL008W
30. YGR159C
31. YGR187C
32. YML093W
33. YHR156C
34. YFL034W
35. YJL123C
36. YNR038W
37. YKL028W
38. YKR048C
39. YLR331C
40. YMR091C
41. YNL186W
42. YDR060W
43. YJR072C
44. YPR152C
45. YBL011W
46. YBL047C
47. YNL099C
48. YDR496C
49. YGL256W
50. YIL138C
51. YBR296C
52. YPL217C
53. YNL254C
54. YLR435W
55. YIR012W
56. YGR202C
57. YFR015C
58. YJL170C
59. YNL079C
60. YKL195W

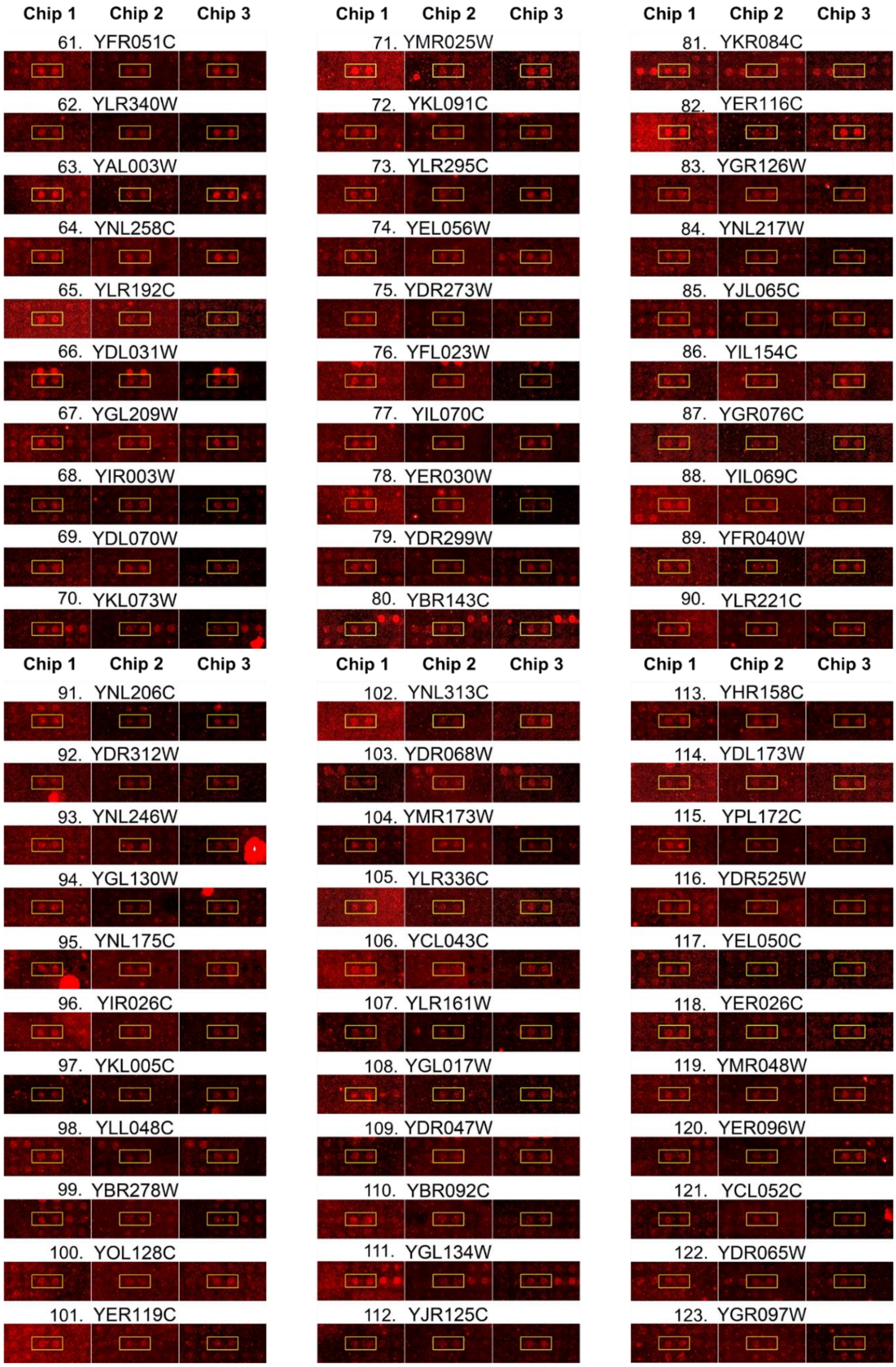

Figure S1. Enlarged protein image of 123 protein targets of penetratin obtained from the triplicate yeast proteome microarrays assay.

Table S2. Entire list of enrichment terms in biological process for the protein targets of penetratin.

Term	Enrichment in Biological Process	*p*-Value	Hit in This Category	Total Gene in This Category
GO:0044085	cellular component biogenesis	0.000041	43	1230
GO:0022613	ribonucleoprotein complex biogenesis	0.000061	24	502
GO:0042254	ribosome biogenesis	0.000074	21	406
GO:0042273	ribosomal large subunit biogenesis	0.000098	11	121
GO:0043933	macromolecular complex subunit organization	0.000099	35	938
GO:0000027	ribosomal large subunit assembly	0.000115	7	41
GO:0071826	ribonucleoprotein complex subunit organization	0.000117	14	202
GO:0022618	ribonucleoprotein complex assembly	0.000259	13	190
GO:0006333	chromatin assembly or disassembly	0.000356	8	70
GO:0071840	cellular component organization or biogenesis	0.000368	69	2611
GO:0042255	ribosome assembly	0.000748	8	79
GO:0006334	nucleosome assembly	0.00103	5	24
GO:0022607	cellular component assembly	0.00103	30	848
GO:0031497	chromatin assembly	0.00116	6	42
GO:0061572	actin filament bundle organization	0.00162	5	27
GO:0051017	actin filament bundle assembly	0.00162	5	27
GO:0048523	negative regulation of cellular process	0.00169	23	592
GO:0031324	negative regulation of cellular metabolic process	0.00197	19	447
GO:0048519	negative regulation of biological process	0.00210	23	602
GO:0009892	negative regulation of metabolic process	0.00212	19	450
GO:0006325	chromatin organization	0.00238	16	346
GO:0010605	negative regulation of macromolecule metabolic process	0.00271	18	423
GO:0007015	actin filament organization	0.00284	7	74
GO:0080090	regulation of primary metabolic process	0.00363	35	1137
GO:0031323	regulation of cellular metabolic process	0.00409	35	1145
GO:0090304	nucleic acid metabolic process	0.00464	47	1709
GO:0060255	regulation of macromolecule metabolic process	0.00573	34	1124
GO:0042274	ribosomal small subunit biogenesis	0.00603	9	143
GO:0031326	regulation of cellular biosynthetic process	0.00609	30	952
GO:0006323	DNA packaging	0.00609	6	61
GO:0016043	cellular component organization	0.00610	59	2324
GO:0019222	regulation of metabolic process	0.00624	35	1175
GO:0032984	macromolecular complex disassembly	0.00633	7	87
GO:2000112	regulation of cellular macromolecule biosynthetic process	0.00641	29	912
GO:0009889	regulation of biosynthetic process	0.00647	30	956
GO:0006368	transcription elongation from RNA polymerase II promoter	0.00706	7	89
GO:0010556	regulation of macromolecule biosynthetic process	0.00734	29	921
GO:0034622	cellular macromolecular complex assembly	0.00851	20	553
GO:0031327	negative regulation of cellular biosynthetic process	0.00910	15	362
GO:0009890	negative regulation of biosynthetic process	0.00931	15	363
GO:0016072	rRNA metabolic process	0.01125	13	297
GO:0046483	heterocycle metabolic process	0.01129	52	2031
GO:0040029	regulation of gene expression, epigenetic	0.01157	9	160
GO:0016070	RNA metabolic process	0.01170	40	1455

GO:0006725	cellular aromatic compound metabolic process	0.01177	52	2035
GO:0030029	actin filament-based process	0.01178	8	129
GO:2000113	negative regulation of cellular macromolecule biosynthetic process	0.01185	14	336
GO:1901985	positive regulation of protein acetylation	0.01211	3	9
GO:0035066	positive regulation of histone acetylation	0.01211	3	9
GO:0034724	DNA replication-independent nucleosome organization	0.01211	3	9
GO:2000758	positive regulation of peptidyl-lysine acetylation	0.01211	3	9
GO:0010558	negative regulation of macromolecule biosynthetic process	0.01270	14	339
GO:0043241	protein complex disassembly	0.01356	6	74
GO:0006348	chromatin silencing at telomere	0.01431	6	75
GO:0000462	maturation of SSU-rRNA from tricistronic rRNA transcript (SSU-rRNA, 5.8S rRNA, LSU-rRNA)	0.01464	7	104
GO:2000756	regulation of peptidyl-lysine acetylation	0.01494	3	10
GO:0035065	regulation of histone acetylation	0.01494	3	10
GO:1901983	regulation of protein acetylation	0.01494	3	10
GO:0065003	macromolecular complex assembly	0.01517	21	627
GO:0006950	response to stress	0.01629	25	802
GO:0051171	regulation of nitrogen compound metabolic process	0.01651	29	979
GO:0006364	rRNA processing	0.01823	12	280
GO:0034728	nucleosome organization	0.01849	6	80
GO:0010468	regulation of gene expression	0.01854	27	899
GO:0006351	transcription, DNA-templated	0.01915	24	770
GO:0034470	ncRNA processing	0.02044	15	400
GO:1901360	organic cyclic compound metabolic process	0.02054	52	2091
GO:0006996	organelle organization	0.02187	44	1704
GO:0050789	regulation of biological process	0.02209	44	1705
GO:0051172	negative regulation of nitrogen compound metabolic process	0.02266	14	366
GO:0044087	regulation of cellular component biogenesis	0.02267	8	147
GO:0030490	maturation of SSU-rRNA	0.02294	7	115
GO:0045934	negative regulation of nucleobase-containing compound metabolic process	0.02301	13	328
GO:0097659	nucleic acid-templated transcription	0.02390	24	786
GO:0006139	nucleobase-containing compound metabolic process	0.02460	49	1961
GO:0071103	DNA conformation change	0.02473	7	117
GO:0070828	heterochromatin organization	0.02495	3	13
GO:0051276	chromosome organization	0.02507	20	617
GO:0032774	RNA biosynthetic process	0.02522	24	790
GO:0030036	actin cytoskeleton organization	0.02661	7	119
GO:0016573	histone acetylation	0.02778	5	60
GO:0006338	chromatin remodeling	0.02790	6	89
GO:0018393	internal peptidyl-lysine acetylation	0.02931	5	61
GO:0006475	internal protein amino acid acetylation	0.02931	5	61
GO:0018394	peptidyl-lysine acetylation	0.02931	5	61
GO:0043412	macromolecule modification	0.02940	29	1026
GO:0044260	cellular macromolecule metabolic process	0.03052	70	3065
GO:0006354	DNA-templated transcription, elongation	0.03066	7	123
GO:0045814	negative regulation of gene expression, epigenetic	0.03107	8	157

GO:0006342	chromatin silencing	0.03107	8	157
GO:0050794	regulation of cellular process	0.03348	42	1651
GO:0006355	regulation of transcription, DNA-templated	0.03545	22	728
GO:1903506	regulation of nucleic acid-templated transcription	0.03638	22	730
GO:2001141	regulation of RNA biosynthetic process	0.03638	22	730
GO:2000617	positive regulation of histone H3-K9 acetylation	0.03814	2	2
GO:0016458	gene silencing	0.03914	8	165
GO:0019219	regulation of nucleobase-containing compound metabolic process	0.03985	24	826
GO:0010629	negative regulation of gene expression	0.04209	12	319
GO:0045892	negative regulation of transcription, DNA-templated	0.04314	11	281
GO:0042787	protein ubiquitination involved in ubiquitin-dependent protein catabolic process	0.04361	4	41
GO:0070925	organelle assembly	0.04364	8	169
GO:1903507	negative regulation of nucleic acid-templated transcription	0.04583	11	284
GO:1902679	negative regulation of RNA biosynthetic process	0.04583	11	284
GO:0031058	positive regulation of histone modification	0.04599	3	18
GO:0016570	histone modification	0.04652	7	136
GO:0022411	cellular component disassembly	0.04652	7	136
GO:0051252	regulation of RNA metabolic process	0.04679	22	750
GO:0051253	negative regulation of RNA metabolic process	0.04958	11	288

Table S3. Comparison of the enrichment terms in the biological process from the four anti-fungal AMPs (Penetratin, Histatin-5, Lfcin B and Sub-5). The corresponding *p*-value of each enriched terms is replaced with the value obtain from –log10(*p*-value).

Enriched terms in Biological process of gene ontology	Penetratin	Histatin-5	LfcinB	Sub-5
cellular component biogenesis	4.387	0	3.199	3.376
ribonucleoprotein complex biogenesis	4.217	3.073	2.345	0
ribosomal large subunit biogenesis	4.010	1.597	0	0
protein-containing complex subunit organization	4.004	0	4.080	1.750
ribonucleoprotein complex subunit organization	3.932	2.369	2.423	0
chromatin assembly or disassembly	3.448	0	0	1.810
cellular component organization or biogenesis	3.434	0	2.234	3.010
cellular component assembly	2.985	0	2.533	2.654
negative regulation of biological process	2.678	0	2.412	2.857
regulation of primary metabolic process	2.440	0	1.916	3.161
nucleic acid metabolic process	2.333	2.106	2.249	1.354
cellular component organization	2.214	0	1.847	2.513
protein-containing complex disassembly	2.199	0	3.304	2.014
transcription elongation from RNA polymerase II promoter	2.151	0	1.383	2.595
heterocycle metabolic process	1.947	1.781	2.170	0
regulation of gene expression, epigenetic	1.937	0	0	0
cellular aromatic compound metabolic process	1.929	1.764	2.150	0
actin filament-based process	1.929	0	1.696	4.848
regulation of protein acetylation	1.826	0	0	0
response to stress	1.788	0	1.751	2.407
rRNA processing	1.739	2.697	1.437	0
transcription, DNA-templated	1.718	0	0	0
organic cyclic compound metabolic process	1.687	1.539	2.093	0
organelle organization	1.660	0	0	2.892
regulation of biological process	1.656	0	1.618	3.264
regulation of cellular component biogenesis	1.645	0	1.883	3.009
DNA conformation change	1.607	0	0	0
chromosome organization	1.601	0	0	1.767
actin cytoskeleton organization	1.575	0	1.380	4.457
macromolecule modification	1.532	0	0	2.496
cellular macromolecule metabolic process	1.515	0	0	0
regulation of cellular process	1.475	0	1.665	3.646
negative regulation of gene expression	1.376	0	0	0
ubiquitin-dependent protein catabolic process	1.360	0	1.959	0
organelle assembly	1.360	0	0	0
cellular component disassembly	1.332	1.383	2.076	1.620
regulation of translation	0	1.593	1.321	0
cellular catabolic process	0	1.609	0	0
negative regulation of catalytic activity	0	1.583	1.911	0
translational initiation	0	1.327	0	0
regulation of cellular amide metabolic process	0	1.977	0	0
posttranscriptional regulation of gene expression	0	1.453	0	0
negative regulation of cellular protein metabolic process	0	0	2.271	0
regulation of protein modification process	0	0	1.596	3.168

DNA-templated transcription, termination	0	0	1.457	0
regulation of cellular component size	0	0	1.303	0
regulation of actin filament-based process	0	0	1.725	0
regulation of protein-containing complex assembly	0	0	1.911	2.568
regulation of molecular function	0	0	1.556	2.678
protein modification by small protein conjugation or removal	0	0	1.774	0
regulation of protein metabolic process	0	0	2.561	2.236
protein ubiquitination	0	0	1.495	0
reproduction	0	0	0	1.968
response to abiotic stimulus	0	0	0	1.302
asexual reproduction	0	0	0	2.819
response to stimulus	0	0	0	1.342
biological regulation	0	0	0	2.996
establishment of cell polarity	0	0	0	1.523
establishment or maintenance of cell polarity	0	0	0	1.457
cytoskeleton organization	0	0	0	4.187
cell division	0	0	0	2.785
cell cycle process	0	0	0	1.538
cell cycle	0	0	0	1.469
phosphorylation	0	0	0	1.684
actin cortical patch organization	0	0	0	3.246
regulation of transferase activity	0	0	0	1.441
regulation of cytokinesis	0	0	0	1.313
protein-DNA complex subunit organization	0	0	0	1.741
regulation of signaling	0	0	0	1.604
regulation of localization	0	0	0	1.341
regulation of response to stimulus	0	0	0	2.800
regulation of signal transduction	0	0	0	1.683
regulation of cell communication	0	0	0	2.063
response to heat	0	0	0	1.692
positive regulation of transcription elongation from RNA polymerase II promoter	0	0	0	1.844
regulation of transcription by RNA polymerase III	0	0	0	1.313
regulation of phosphate metabolic process	0	0	0	2.340
regulation of phosphorus metabolic process	0	0	0	2.340
regulation of DNA-templated transcription, elongation	0	0	0	1.580
protein polymerization	0	0	0	1.325
